# Supplementary material for: Rechargeable potassium-ion batteries with honeycomb-layered tellurates as high voltage cathodes and fast potassium-ion conductors
Source: Nat Commun. 2018 Sep 20;9:3823. doi: 10.1038/s41467-018-06343-6 (PMC6147795; doi:10.1038/s41467-018-06343-6)
Supplement: Supplementary file 1 — Supplementary Information (PDF file) [file 41467_2018_6343_MOESM1_ESM.pdf]

Supplementary Information

Rechargeable Potassium-Ion Batteries with Honeycomb-Layered Tellurates Inducing New High Voltage Cathodes and Fast Potassium-Ion Conductors

T. Masese *et al*.

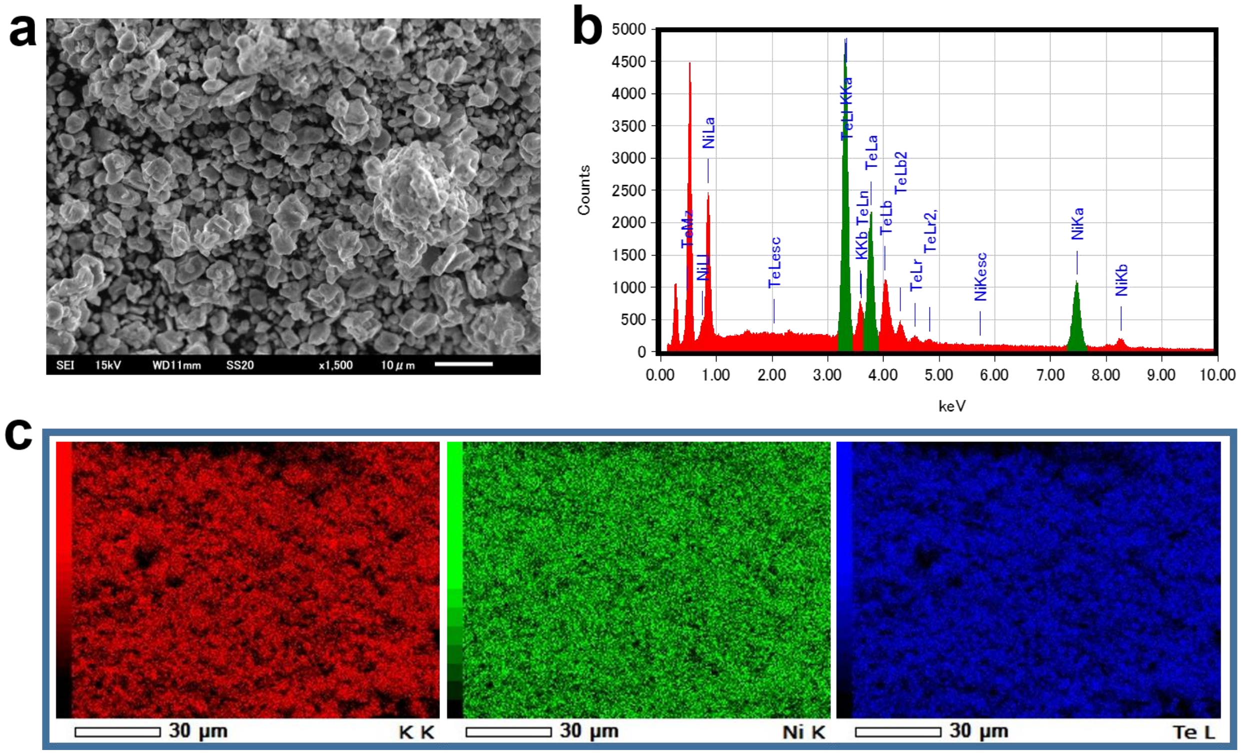

Supplementary Figure 1. a. SEM picture of the $K_2Ni_2TeO_6$ powders and **b.** the SEM-EDX spectrum of the powders, while **c.** shows the elemental mapping of $K_2Ni_2TeO_6$. The general flake-like grain morphology that is usually seen in some lamellar K_xMO_2 (M = Co, Mn) compounds is also evident here.

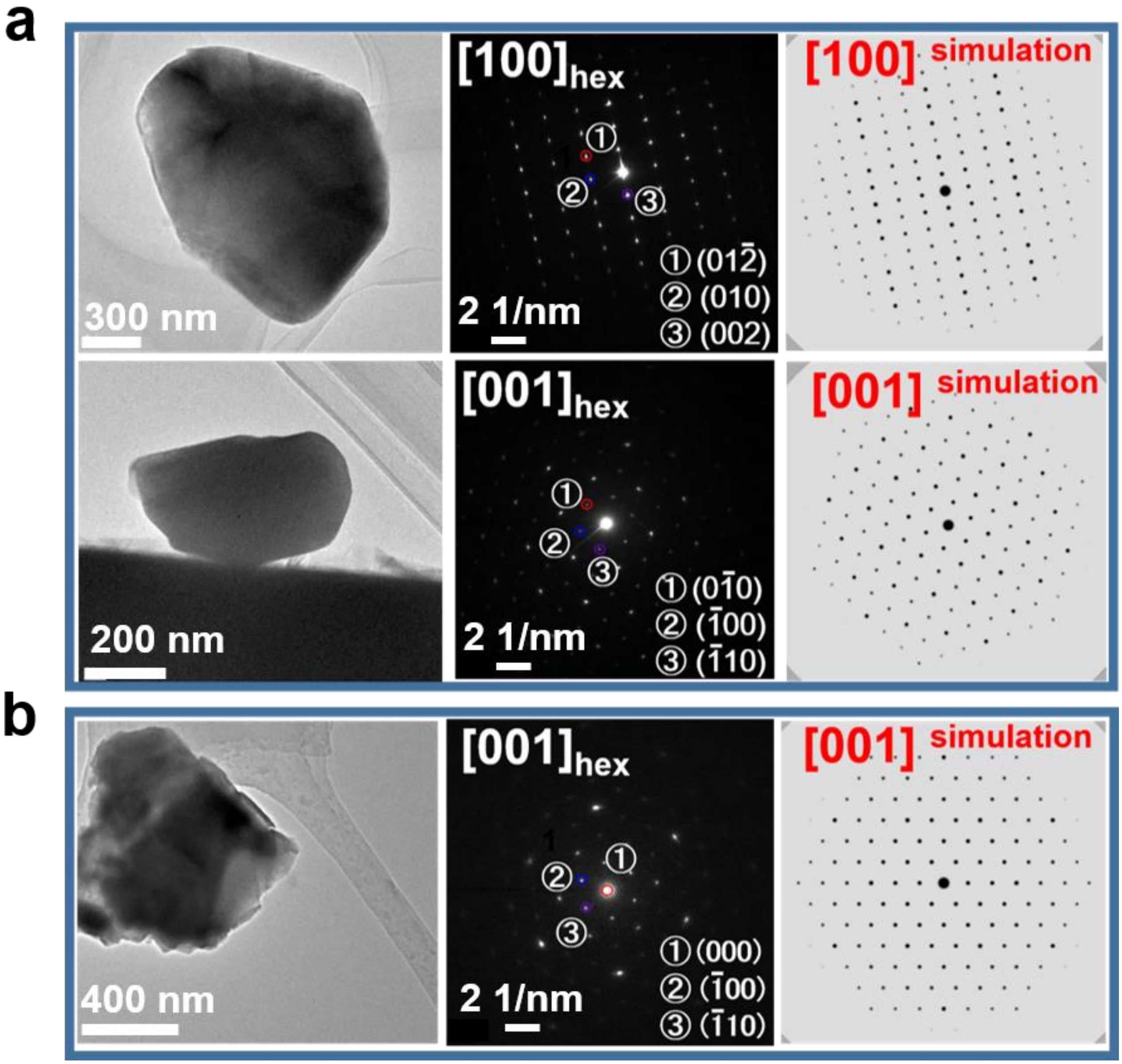

Supplementary Figure 2. a. Electron diffraction patterns recorded on $K_2Ni_2TeO_6$ indexed in a hexagonal lattice along [100] and [001] directions, and **b.** $K_2Mg_2TeO_6$ along [001] zone axis.

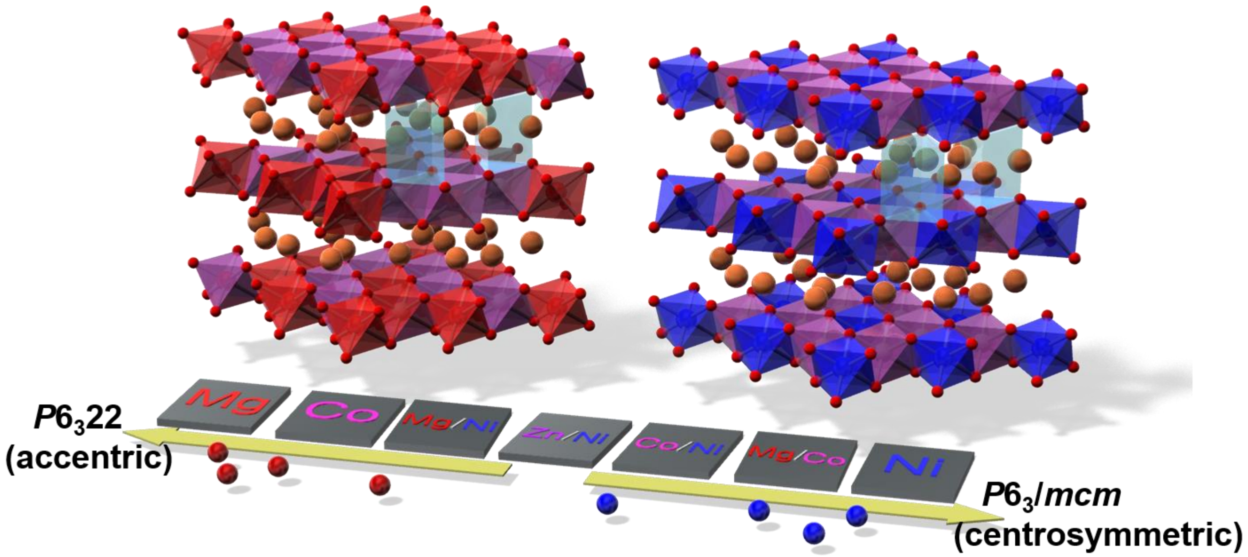

Supplementary Figure 3. Crystal structure transition of potassium orthotellurate family described by the formula $K_2M_2TeO_6$ and $K_2M_{1-x}M'_xTeO_6$, (in which *M* and *M*' are Co, Ni, Zn or Mg). A comparison between the structure of $K_2M_2TeO_6$ (*M* = Ni, Mg, Co) and solid-solution derivatives evinces the stacking difference with distinct atom sequences along the hexagonal *c*-axis. For $K_2Ni_2TeO_6$ end-phase member crystallising in $P6_3/mcm$ space group, both Te and Ni atoms of one slab are located exactly above Te and Ni atoms of the last slab, whereas Te and Mg atoms are alternatively located along the hexagonal *c*-axis for $P6_322$ space group as adopted by $K_2Mg_2TeO_6$ end-phase member

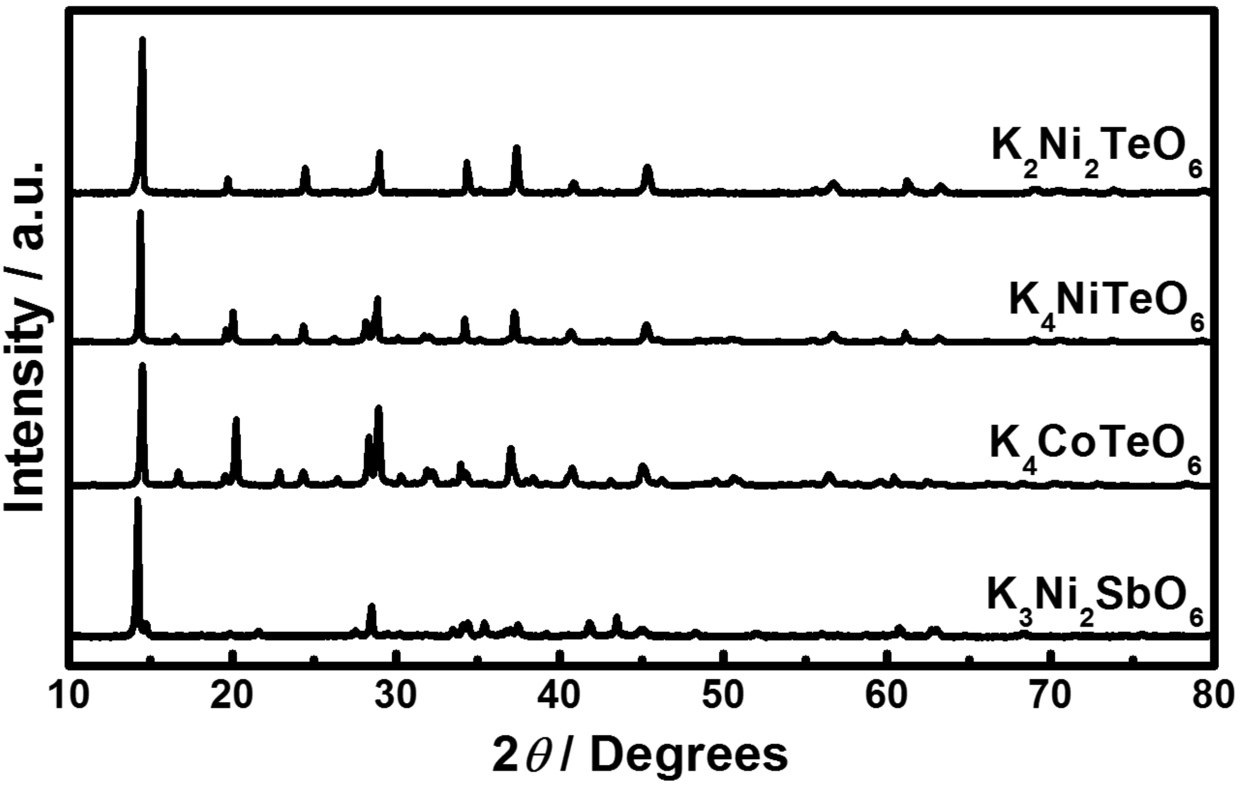

Supplementary Figure 4. Conventional XRD patterns of other derivatives of $K_2Ni_2TeO_6$ (namely, K_4*M*TeO_6 (*M* = Ni, Co) and $K_3Ni_2SbO_6$ (with impurities)) prepared via a conventional solid state ceramics route at 800 °C, as described in the **METHODS** section. K_4*M*TeO_6 are isostructural with Na_4NiTeO_6 honeycomb layered phases. K_4NiTeO_6 crystallises in a monoclinic structure with lattice parameters *a* = 7.26 Å, *b* = 10.08 Å, *c* = 8.65 Å, =114.14º (V = 574.6 Å^3). Detailed structural analyses of K_4*M*TeO_6 (*M* = Ni, Co, Mg, Zn) and phase-pure $K_3Ni_2SbO_6$ are beyond the scope of the current study. Synchrotron XRD studies complemented with TEM analyses are subject of future work. Nonetheless, the present results show that the layered $K_2Ni_2TeO_6$ materials can be extended to encompass a plethora of new stoichiometries, presumably adopting the honeycomb layering scheme seen in the orthotellurate compounds.

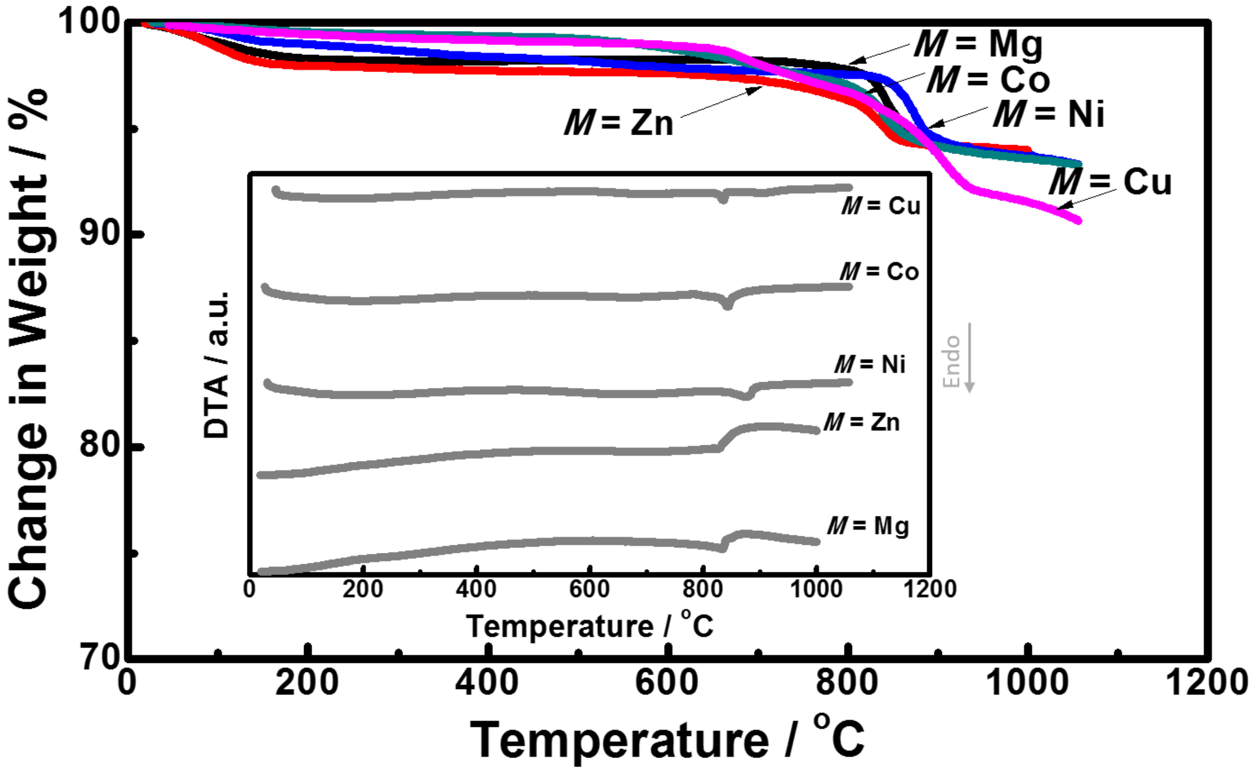

Supplementary Figure 5. Thermogravimetry (TG) and differential thermal analysis (DTA) curves (inset) of $K_2M_2TeO_6$ (M = Ni, Co, Mg, Cu and Zn) ranging from 25 to 1000 °C. The onset of an endothermic peak at the DTA curve (around 800~900 °C) indicates the melting point temperature of $K_2M_2TeO_6$. The weight loss observed at around 100 °C arises from water absorbed during the loading of the powders in Pt ampoules prior to measurements.

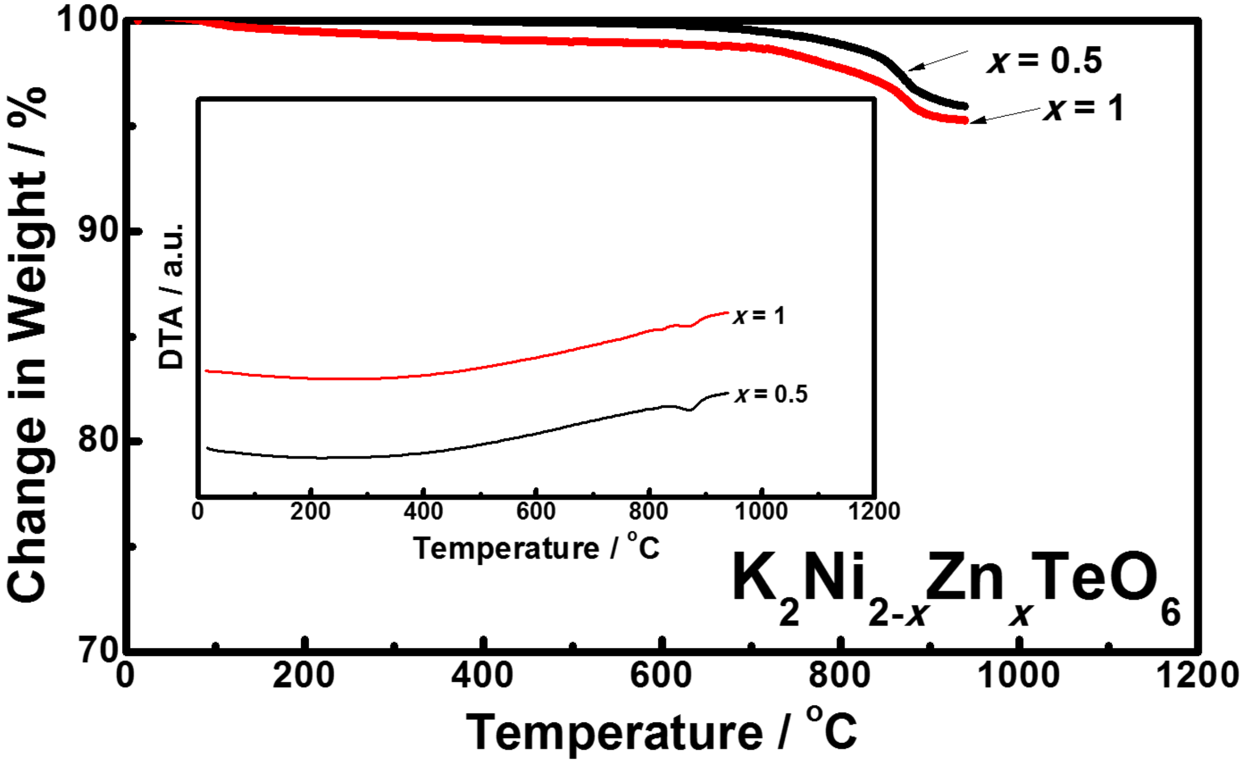

Supplementary Figure 6. Thermogravimetry (TG) and differential thermal analysis (DTA) curves (inset) of $K_2Ni_{2-x}Zn_xTeO_6$ (x = 0.5 and 1) ranging from 25 to 950 °C.

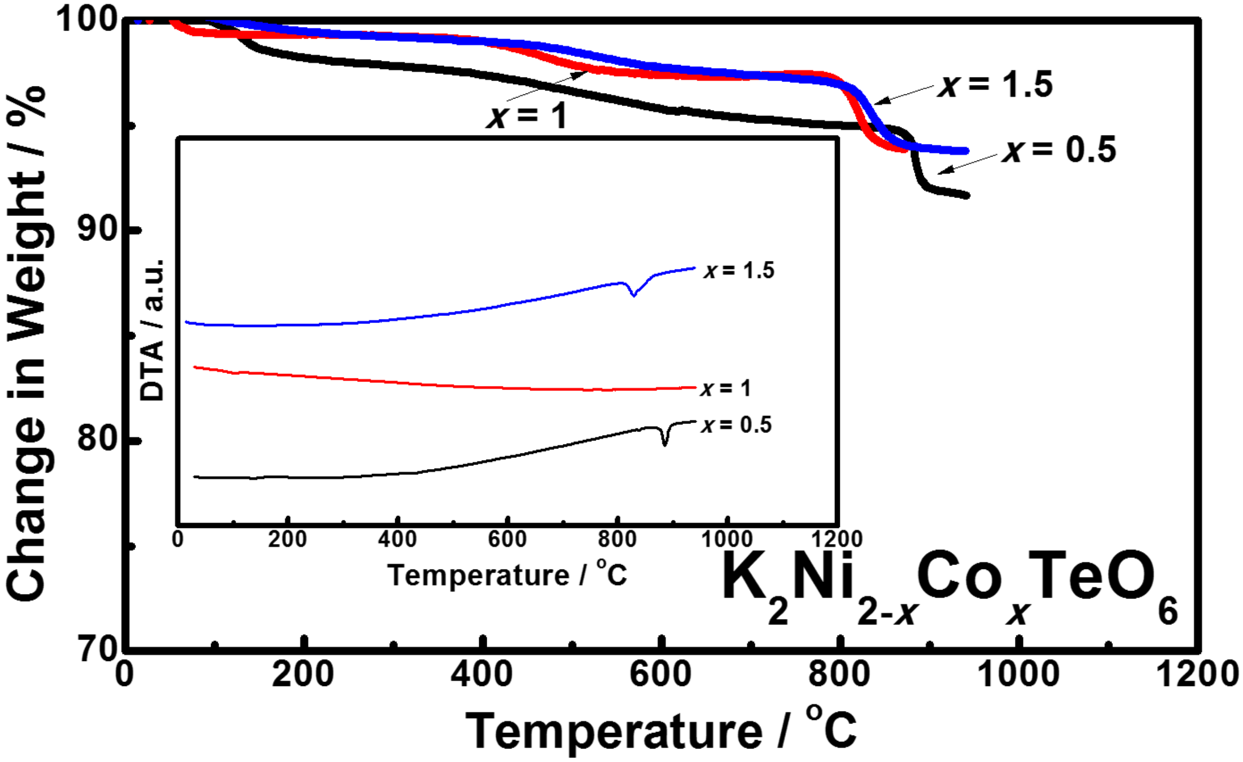

Supplementary Figure 7. Thermogravimetry (TG) and differential thermal analysis (DTA) curves (inset) of $K_2Ni_{2-x}Co_xTeO_6$ (x = 0.5, 1.0 and 1.5) ranging from 25 to 950 °C.

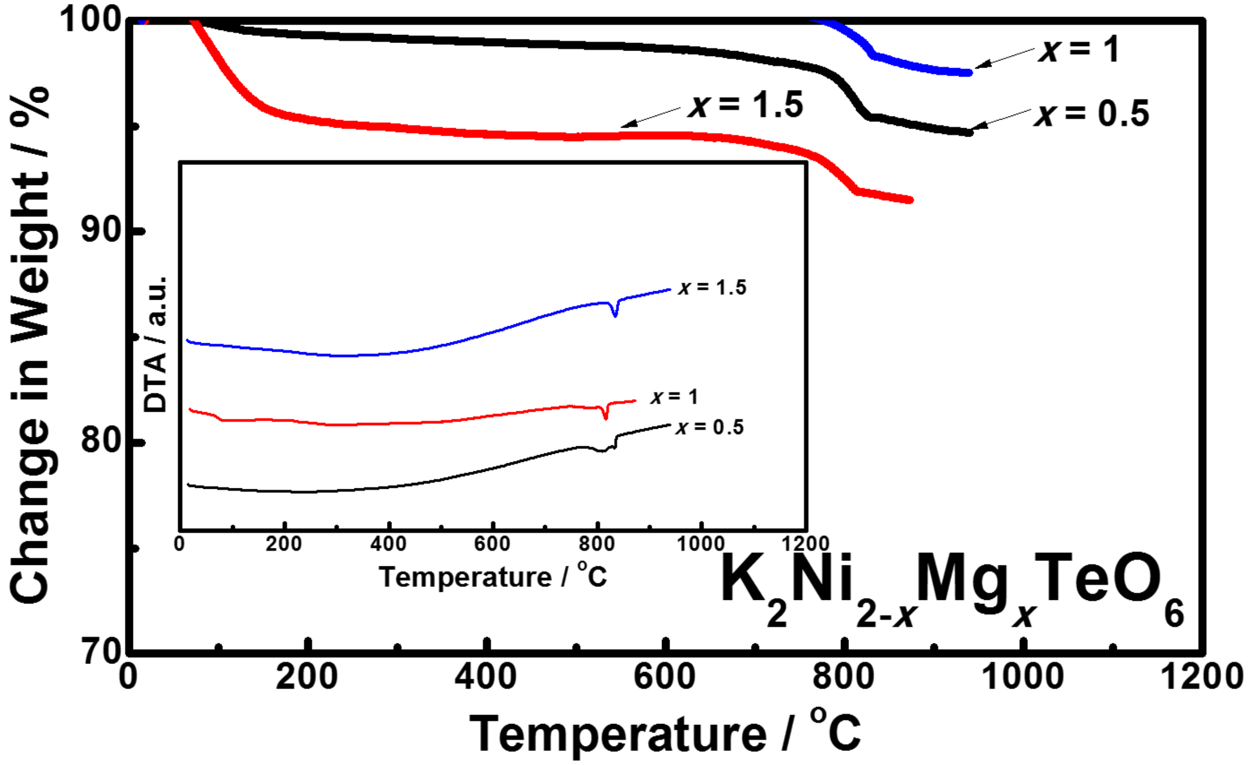

Supplementary Figure 8. Thermogravimetry (TG) and differential thermal analysis (DTA) curves (inset) of $K_2Ni_{2-x}Mg_xTeO_6$ (x = 0.5, 1.0 and 1.5) ranging from 25 to 950 °C.

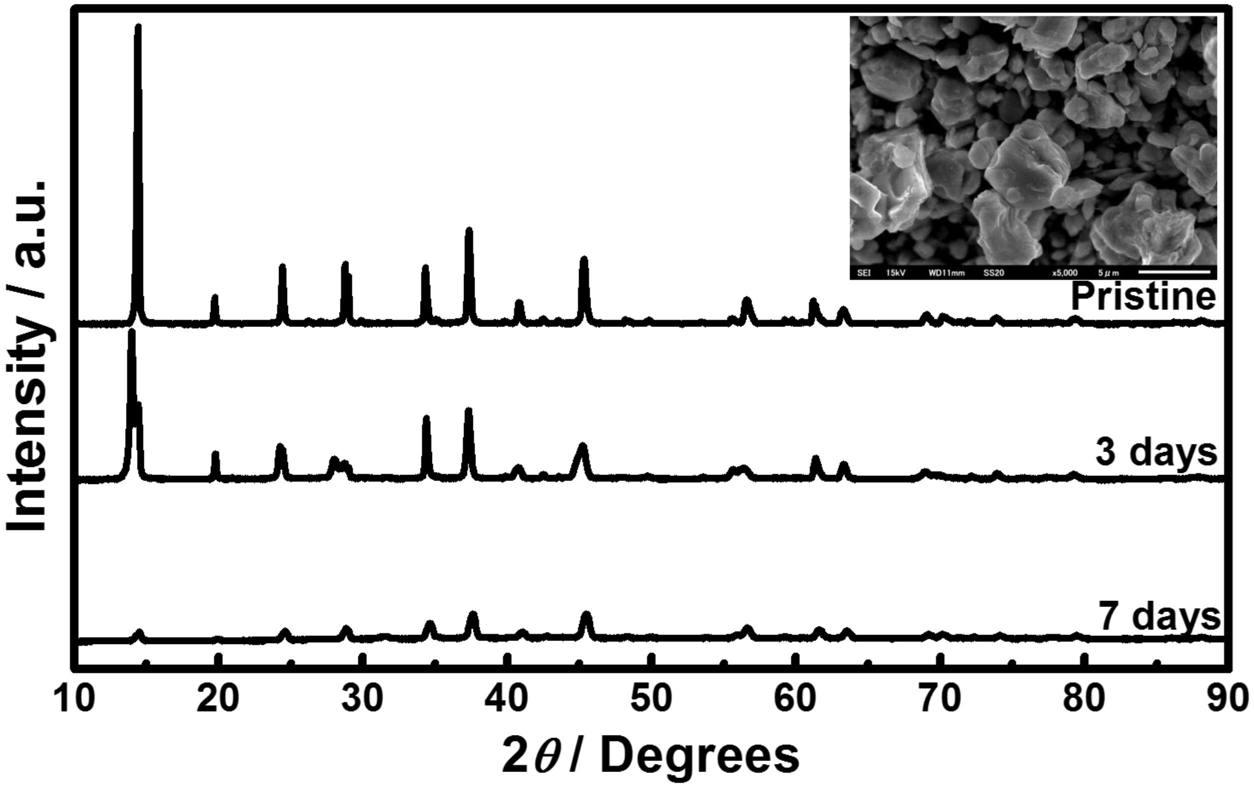

Supplementary Figure 9. Conventional XRD patterns of pristine $K_2Ni_2TeO_6$, revealing a diminution in the intensity of diffraction peaks upon moist air exposure with a relative humidity in the range of 70 ~ 75 %. $K_2Ni_2TeO_6$ needs to be handled in dry inert atmosphere owing to the propensity to amorphise (or undergo phase change) upon exposure to moist air.

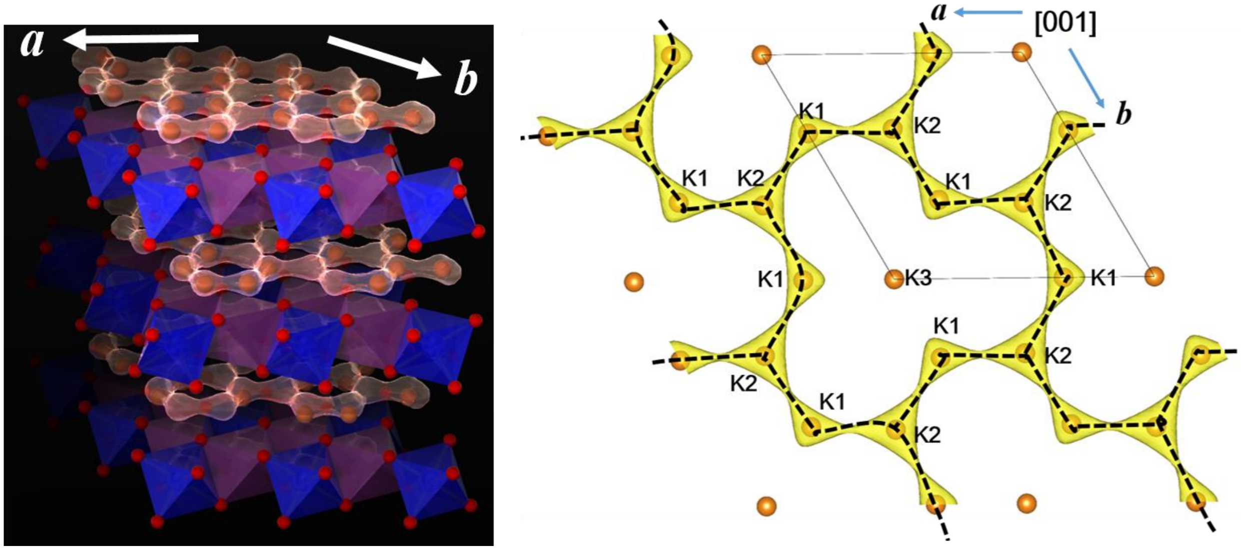

Supplementary Figure 10. a. Schematic presentation of $K_2Ni_2TeO_6$ viewed along [110] direction. NiO_6 tetrahedra (purple), TeO_6 octahedra (blue), and K atoms (brown) are illustrated. **b.** 2D bond valency energy landscape (BVEL) map of $K_2Ni_2TeO_6$ (yellow) with isosurfaces of 0.35 eV for K depicting a circular K-ion diffusion pattern (akin to a rotary Wankel-type motor engine).

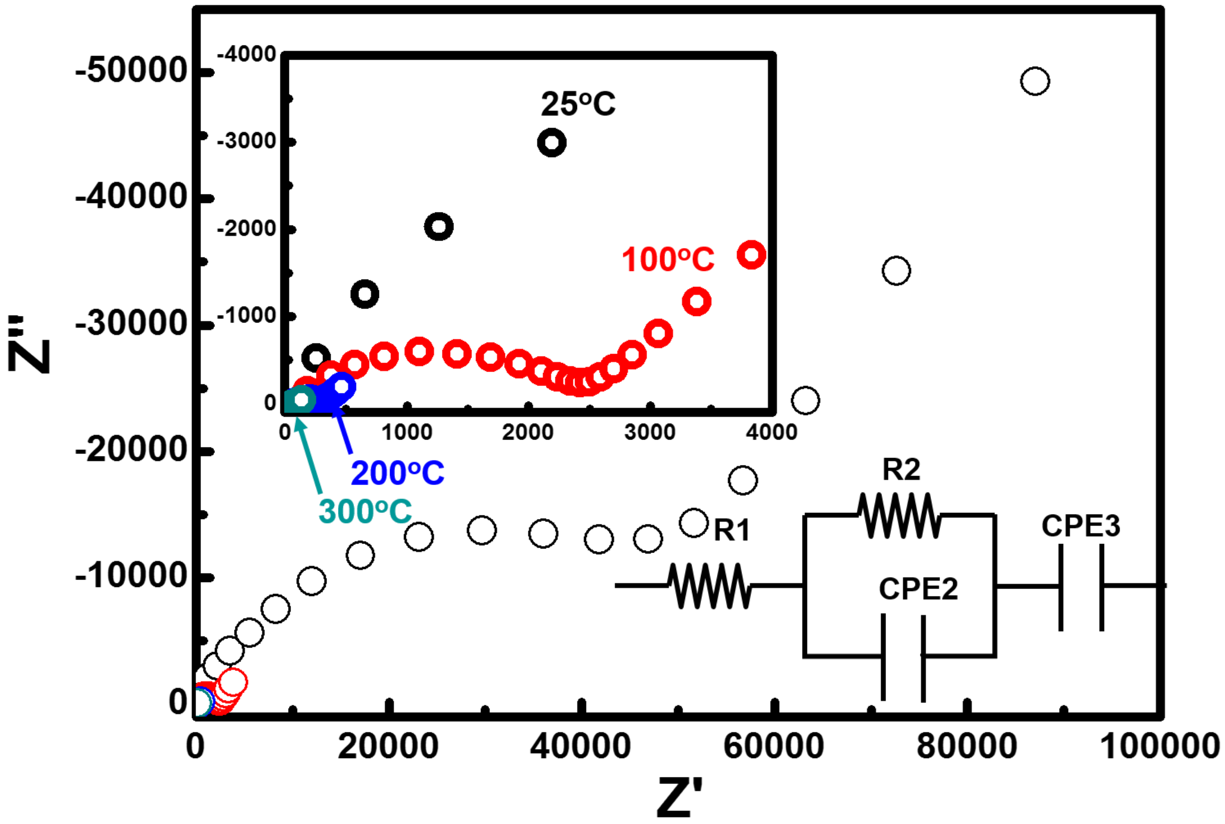

Supplementary Figure 11. Complex a.c. impedance spectra (Nyquist plots) obtained for $K_2Mg_2TeO_6$ at various temperatures (namely, 25, 100, 200 and 300 °C). A semicircle can be observed at high frequencies, which can be attributed to the solid electrolyte potassium ion conduction. The spectra were fitted by an equivalent circuit (shown in inset figure) composed of an initial resistor *R*1 (for the device and current collectors' resistances) in series with one resistor *R*2 (for solid electrolyte resistance) in parallel with a constant phase element CPE2 (for the non-ideal capacitance between particles) and in series with another CPE3 (for the impedance of both the top and bottom electrode-electrolyte junctions). The ionic conductivity (σ) was then calculated using the equation $\sigma = l / RS$ in which l is the thickness of the solid electrolyte pellet, while S denotes the pellet surface area and R is the resistance obtained from the fitted $R2$ value. A drastic diminution of the semicircle diameter is noticeable upon heating, in accord with the enhancement of the ionic conductivity with temperature.

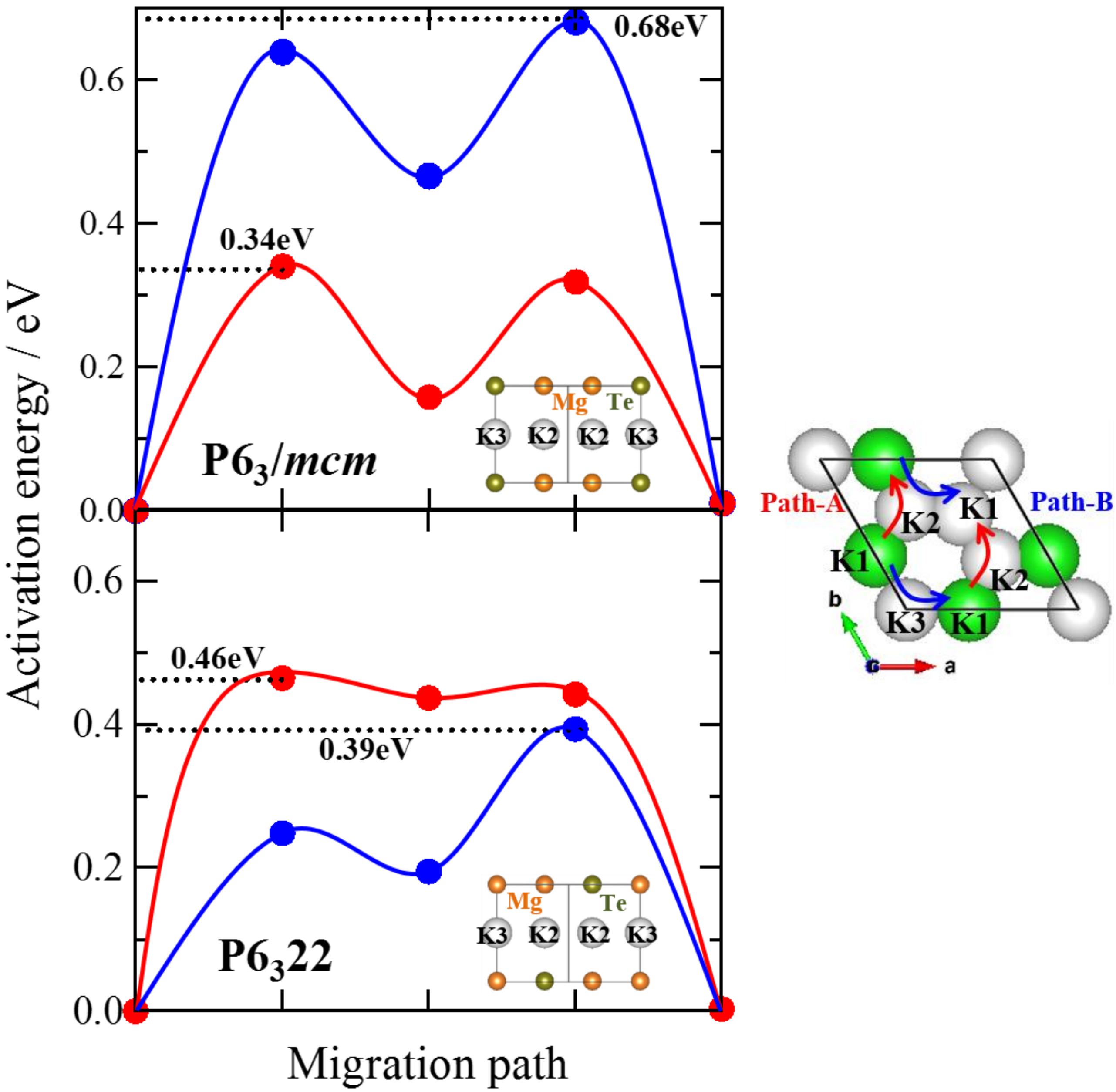

Supplementary Figure 12. Calculated K^+ migration activation energies along the pathways A and B in $K_2Mg_2TeO_6$. The green and white balls represent, respectively, occupied and vacant K^+ sites in the initial model before migration. Cooperative diffusion pathways formed by correlated two K^+ ions at K1 sites via K2+K2 sites (Path-A; red) or via K2+K3 sites (Path-B; blue) are schematically drawn. Schematic structures at ½ *c* size on the [110] planes including Mg and Te ions are inserted.

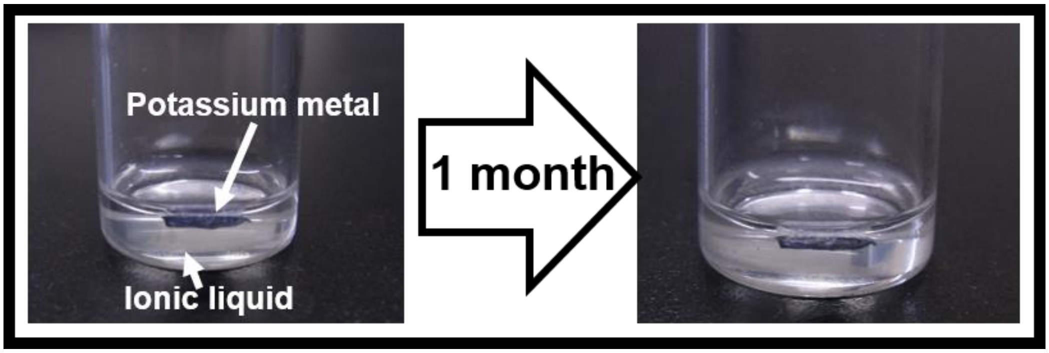

Supplementary Figure 13. Stability of (0.5 M KTFSI in Pyr_{13}TFSI) ionic liquid against potassium (K) metal after one month. The colour does not change, indicating no decomposition of electrolyte in the presence of potassium. This further confirms the stable electrochemical performance observed.

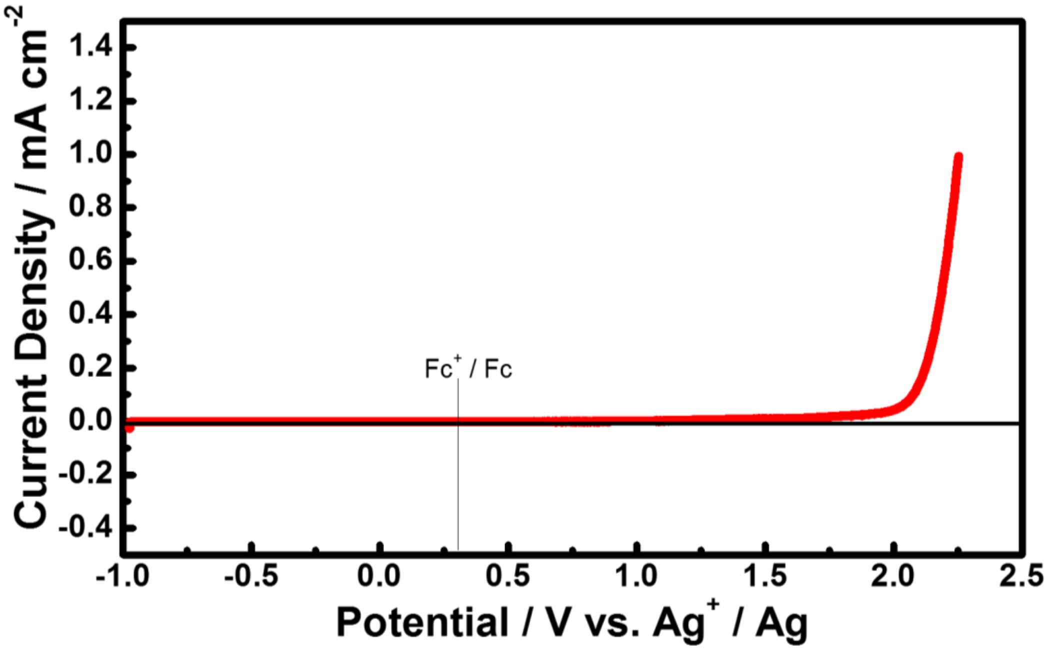

Supplementary Figure 14. Linear sweep voltammogram of Pt electrode in $Pyr_{13}TFSI$ ionic liquid containing 0.5 M KTFSI using three-electrode cells. Pt was used as the counter electrode. Reference electrode was silver wire immersed in $Pyr_{13}TFSI$ containing 0.1 M $AgCF_3SO_3$. Potential sweep rate was set at 1.0 mV s^{-1}, and measurements were conducted at 25 °C. Ionic conductivity of the 0.5 M KTFSI in $Pyr_{13}TFSI$ ionic liquid was 1.8 mS cm^{-1} at 25 °C.

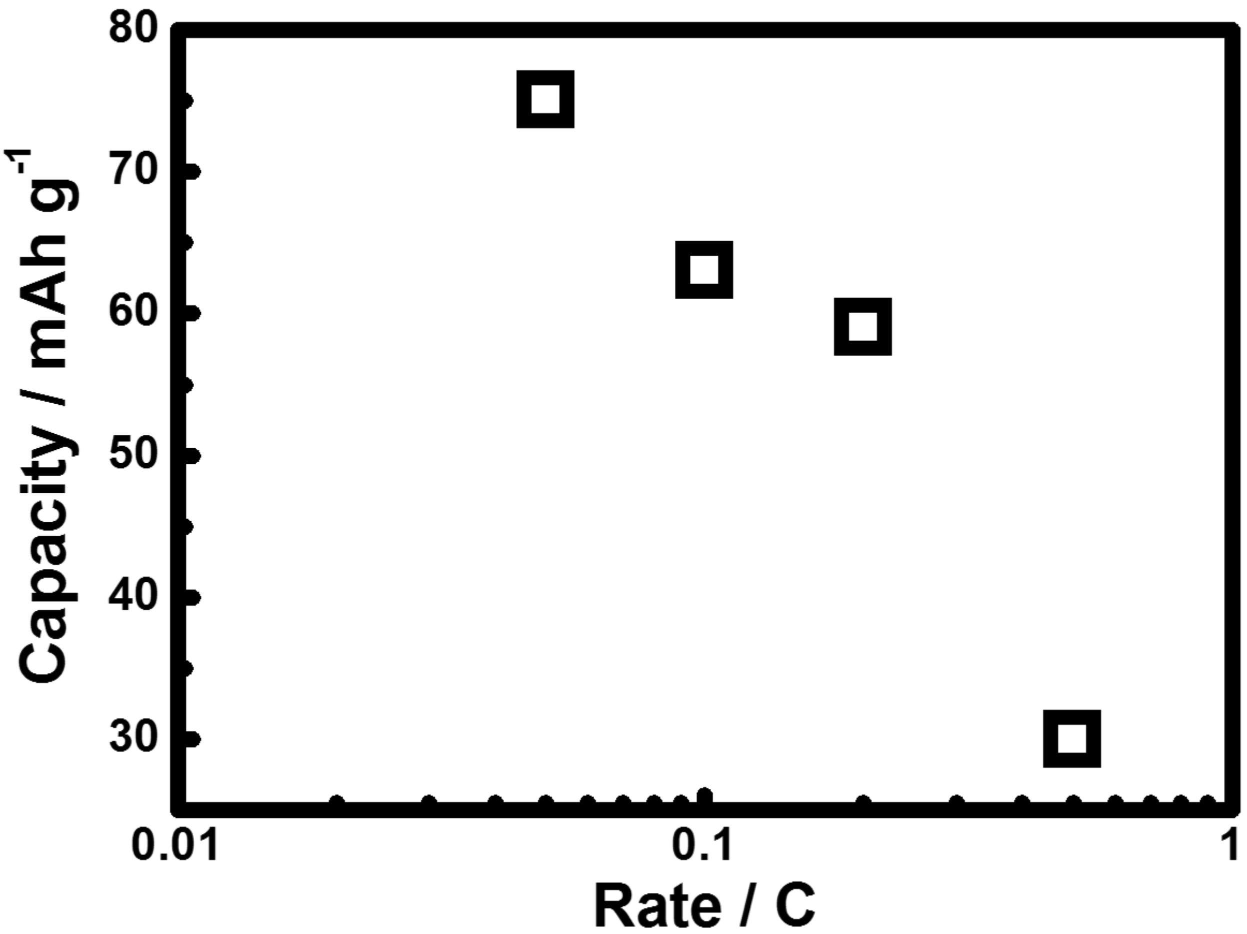

Supplementary Figure 15. Rate performance of $K_2Ni_2TeO_6$ in K half-cells using 0.5 M KTFSI in Pyr_{13}TFSI ionic liquid. Galvanostatic measurements were conducted at various rates at room temperature.

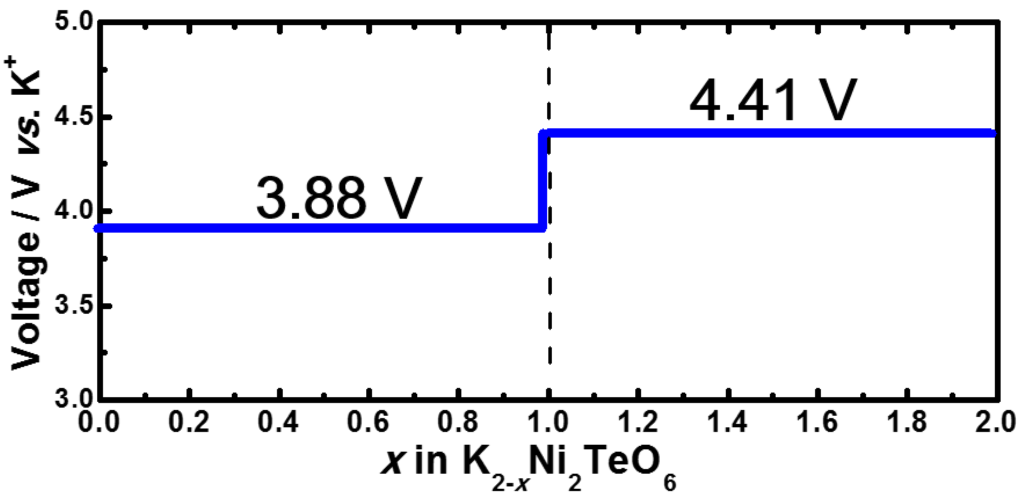

Supplementary Figure 16. Calculated voltages of $K_2Ni_2TeO_6$ upon K-ion extraction.

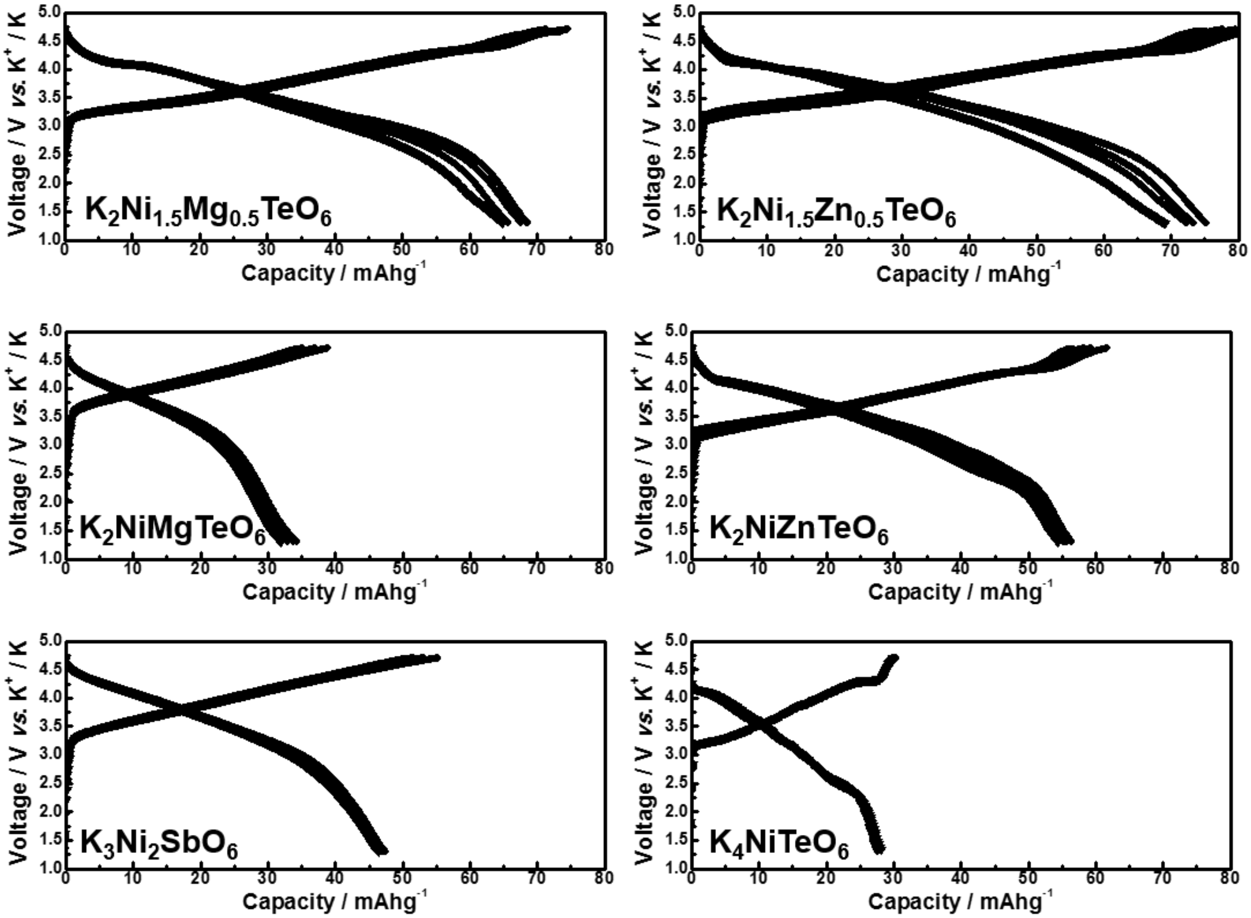

Supplementary Figure 17. Voltage-capacity profiles (20 cycles) for $K_2Ni_2TeO_6$ solid solutions in K half-cells using 0.5 M KTFSI in $Pyr_{13}TFSI$ ionic liquid. Galvanostatic measurements were conducted at C/20 rate at room temperature. For the sake of readability, the 1st, 5th, 10th and 20th voltage profiles have been shown for $K_2Ni_{1.5}Mg_{0.5}TeO_6$ and $K_2Ni_{1.5}Mg_{0.5}TeO_6$.

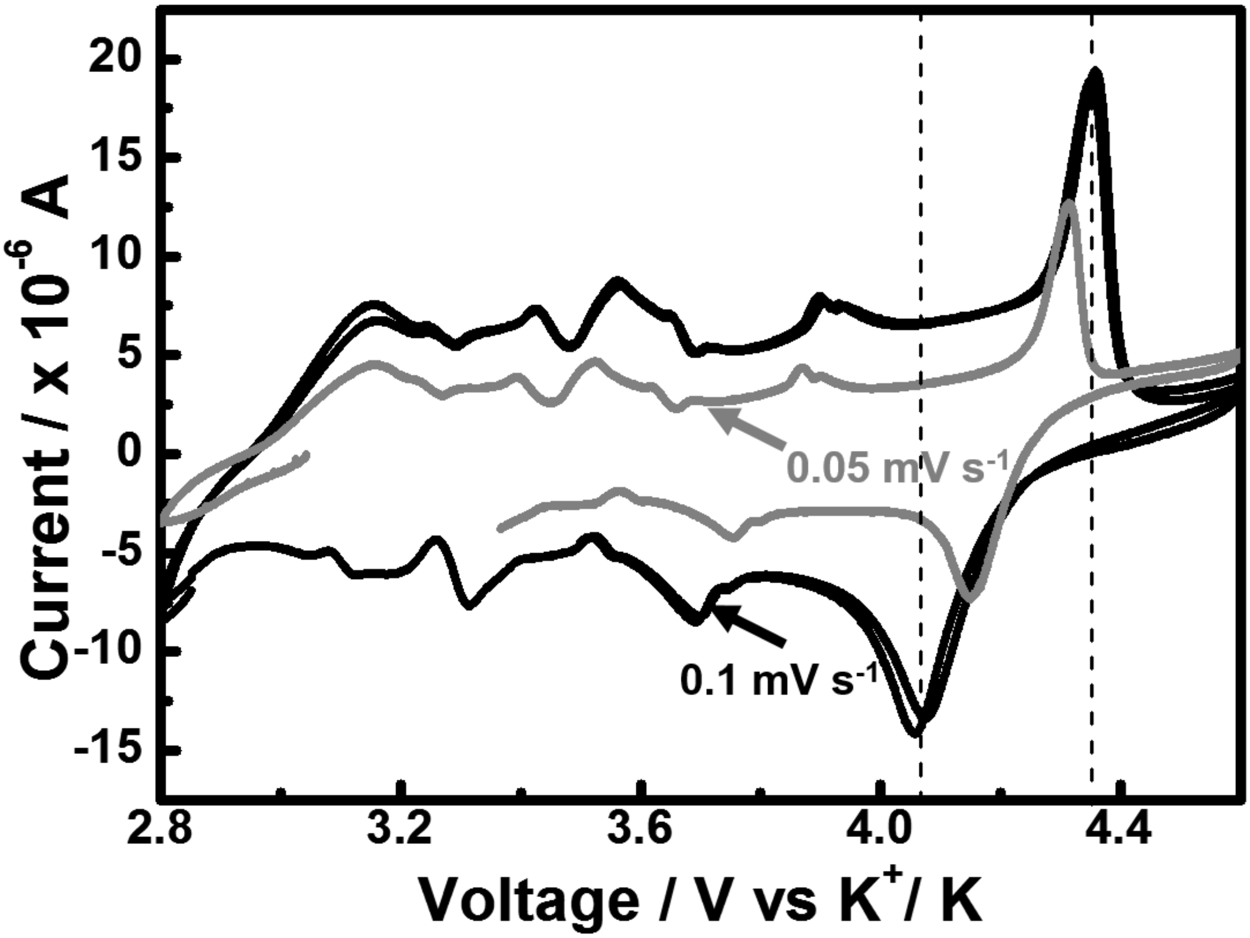

Supplementary Figure 18. Cyclic voltammograms of $K_2Ni_2TeO_6$ taken at a scanning rate of 0.05 mV s^{-1} (grey curve) and 0.1 mV s^{-1} (black curve) revealing redox voltages above 4 V versus (*vs*.) K^+ / K. Cyclic voltammograms of $K_2Ni_2TeO_6$ taken at a scanning rate of 0.1 mV s^{-1} revealing redox voltages above 4 V vs. K^+ / K. Minor peaks emanating from a multitude of K^+ / vacancy ordering as has been noted in P2-type structures during K-ion (de)insertion are also evident.

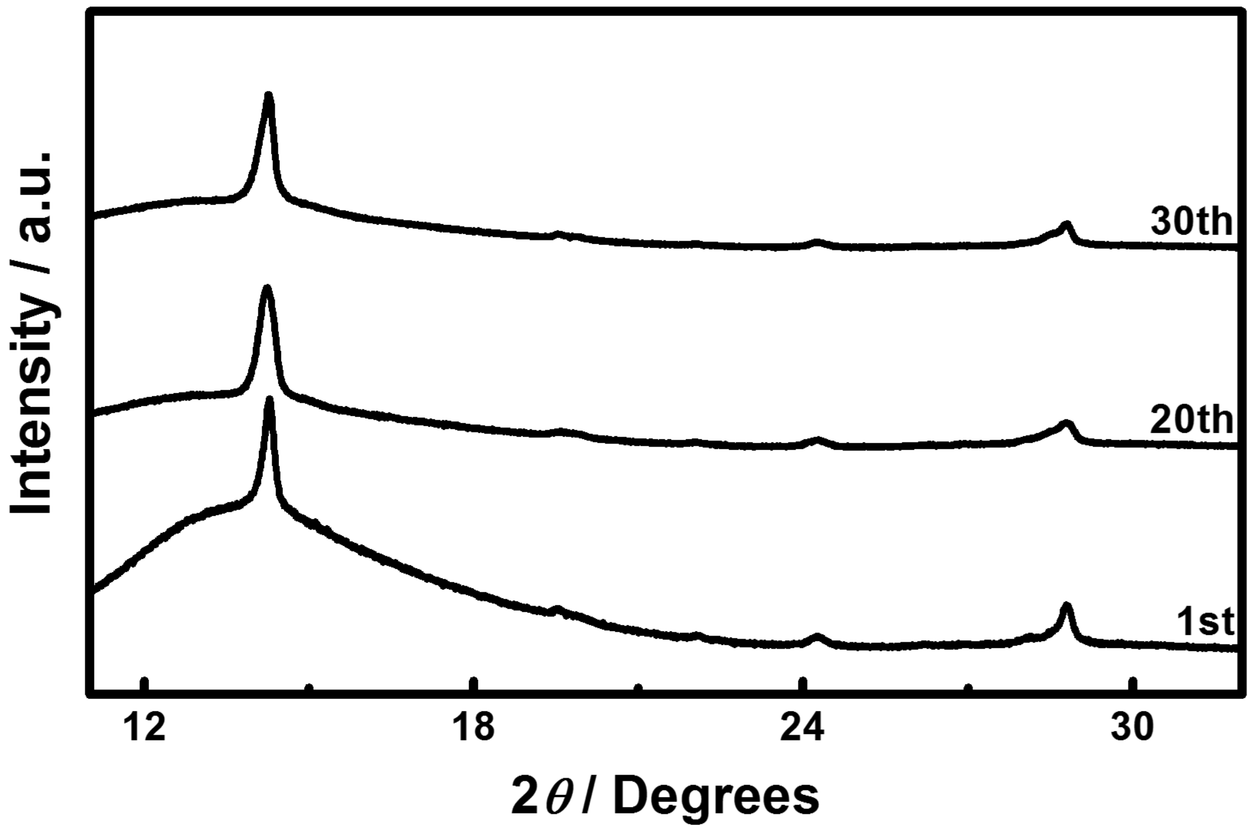

Supplementary Figure 19. X-ray diffraction *ex situ* patterns of $K_2Ni_2TeO_6$ taken upon initial, 20th and 30th cycling. No discernible change in the XRD powder pattern was found for samples that had been cycled for more than 20 and 30 cycles, which is consistent with a sustained reversibility of the P2-type $K_2Ni_2TeO_6$ upon repeated K^+ extraction / insertion.

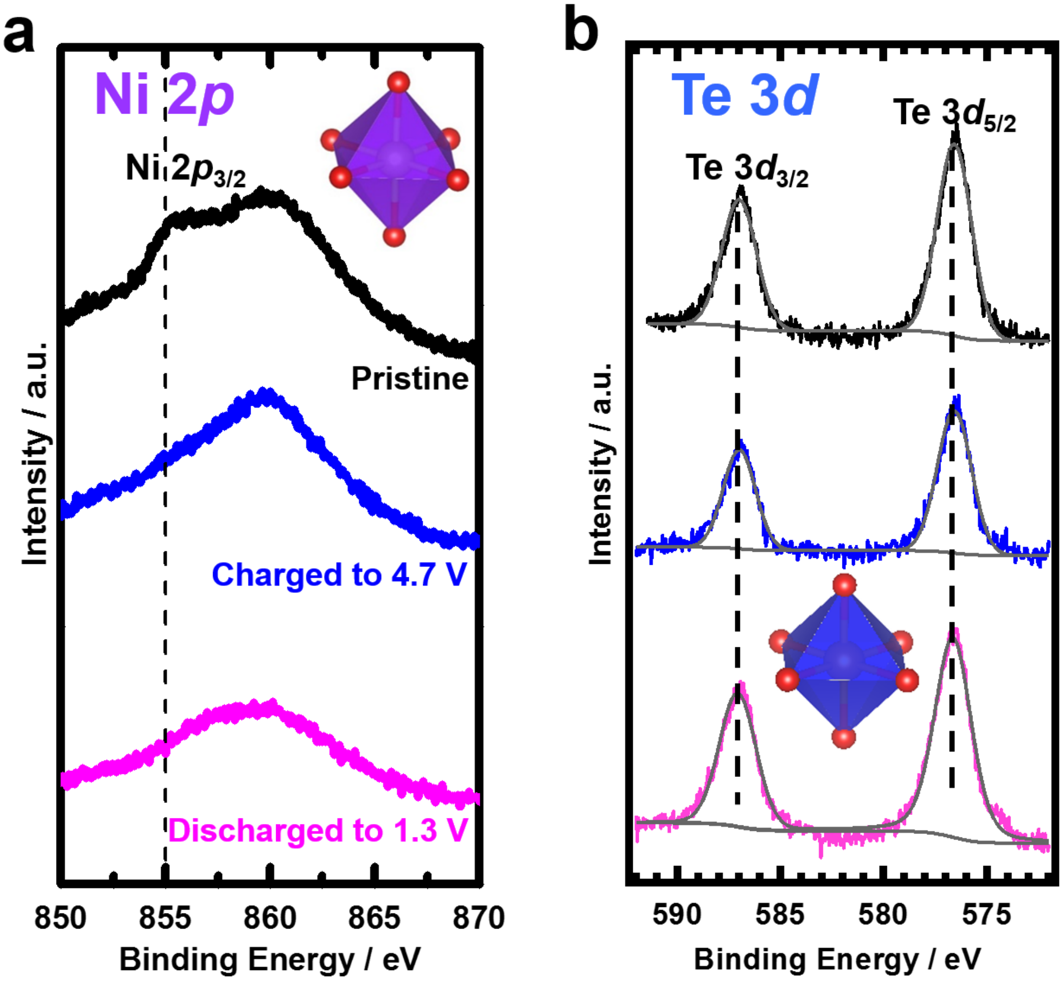

Supplementary Figure 20. a. Ni 2$p_{3/2}$ and **b.** Te 3*d* XPS spectra of $K_2Ni_2TeO_6$ taken after charging to 4.7 V and discharging to 1.3 V. The broad peak (hump) appearing at around 860 eV shows the F(KL_1L_{23}) Auger peak. The binding energy of the Ni 2$p_{3/2}$ peak shifts to a higher value of 857 eV, which corresponds to Ni^{3+} after charging to 4.6 V. After discharging, the Ni 2$p_{3/2}$ peak recovers to the shape of pristine phase, confirming mainly the reversibility of the process in agreement with the electrochemical data. Turning to Te 3*d* core spectra, the Te 3$d_{5/2}$ peak at 576.3 eV is characteristic of Te^{6+} referenced to K_2TeO_3 (Te 3$d_{5/2}$ respectively at 576.3 and 575.7 eV). It remains invariant for the entire charge–discharge process indicating that Te, as a spectator ion, does not participate in the redox process.

Supplementary Table 1. Results of Inductively Coupled Plasma (ICP) measurements of $K_2M_2TeO_6$ (M = Ni, Co, Mg).

**Compound**	**K**	***M***	**Te**
	**(molar ratio)**	**(molar ratio)**	**(molar ratio)**
$K_2Ni_2TeO_6$	1.91	2.01	1
$K_2Co_2TeO_6$	1.70	1.86	1
$K_2Mg_2TeO_6$	1.89	1.95	1

Supplementary Table 2. Atomic coordinates (*x*, *y*, *z*), occupancies (*g*), and isotropic atomic displacement parameters (U_{iso}) obtained by Rietveld refinement of synchrotron X-ray diffraction data for as-prepared $K_2Ni_2TeO_6$ indexed in the space group $P6_3/mcm$ (hexagonal) with lattice constants $a = b = 5.2606(1)$ Å, $c = 12.4669(3)$ Å, and $V = 298.79(1)$ Å^3 ($R_{Bragg} = 4.29$ %, $\chi^2 = 1.77$). The crystal structure of $K_2Ni_2TeO_6$ was solved using the crystal structure of isotypic $Na_2Ni_2TeO_6$ as a starting structural model. The K1, K2 and K3 sites, respectively, have multiplicities of three, two, and one at each interlayer per unit cell. Further details of the crystal structure investigation(s) may be obtained from the Fachinformationszentrum Karlsruhe, D-76344 Eggenstein-Leopoldshafen (Germany), on quoting the depository (accession) numbers CSD-434032, -434033, and -434061.

Atom	Wyckoff	*x*	*y*	*z*	*g*	U_{iso}
Te1	2*b*	0	0	0	1	0.0035(8)
Ni1	4*d*	2/3	1/3	0	1	0.0013(4)
O1	12*k*	0.6723(6)	0.6723(6)	0.5924(3)	1	0.0010(2)
K1	6*g*	0.3495(7)	0	1/4	0.4984(43)	0.0213(7)
K2	4*c*	1/3	2/3	1/4	0.2061(35)	0.0025(5)
K3	2*a*	0	0	1/4	0.0452(21)	0.0709(13)

* *g* and U_{iso} denote the occupancy and isotropic thermal factor, respectively.

Supplementary Table 3. Comparison of the lattice parameters of the potassium orthotellurate family described by the formula $K_2M_2TeO_6$ and $K_2M_{1-x}M'_xTeO_6$, (in which *M* and *M*' are Co, Ni, Zn, Mg, Mn or Cu). Subject to the requirement of charge balance, practically any combination of such solid solutions can be realised. Lattice parameters for solid solution derivatives were determined through Le Bail fitting procedure. Detailed structural analyses of $K_2Zn_2TeO_6$, $K_2Mn_2TeO_6$ and $K_2Cu_2TeO_6$ parent phases will be reported elsewhere.

Compound	Lattice	Symmetry	a_{hex} (=b) (Å)	c_{hex} (Å)	V (Å^3)	ρ (g cm^{-3})
$K_2Co_2TeO_6$	Hexagonal	$P6_322$	5.2426(7)	12.4162(14)	295.54(4)	4.6983
$K_2Mg_2TeO_6$	Hexagonal	$P6_322$	5.2863(5)	12.5388(20)	303.45(6)	4.5758
$K_2Ni_2TeO_6$	Hexagonal	$P6_3/mcm$	5.2606(1)	12.4669(3)	298.79(1)	4.6473
$K_2Co_{1.5}Ni_{0.5}TeO_6$	Hexagonal	$P6_3/mcm$	5.2415(2)	12.5196(9)	297.88(2)	4.6777
$K_2CoNiTeO_6$	Hexagonal	$P6_3/mcm$	5.1952(11)	12.5692(6)	293.79(1)	4.7413
$K_2Co_{0.5}Ni_{1.5}TeO_6$	Hexagonal	$P6_3/mcm$	5.2075(1)	12.5252(5)	294.15(1)	4.7342
$K_2NiZnTeO_6$	Hexagonal	$P6_3/mcm$	5.2691(4)	12.5072(15)	300.71(5)	4.7036
$K_2Ni_{1.5}Zn_{0.5}TeO_6$	Hexagonal	$P6_3/mcm$	5.2651(3)	12.4905(13)	299.87(4)	4.6797
$K_2Co_{0.5}Mg_{1.5}TeO_6$	Hexagonal	$P6_3/mcm$	5.2556(12)	12.6568(53)	302.76(1)	4.0337
$K_2Co_{1.5}Mg_{0.5}TeO_6$	Hexagonal	$P6_3/mcm$	5.2558(2)	12.5386(12)	299.96(3)	4.4548
$K_2NiMgTeO_6$	Hexagonal	$P6_322$	5.5034(11)	12.0903(18)	317.13(7)	4.0311
$K_2Ni_{1.5}Mg_{0.5}TeO_6$	Hexagonal	$P6_3/mcm$	5.4665(10)	12.1301(12)	313.91(6)	4.2544
$K_2Ni_{0.5}Mg_{1.5}TeO_6$	Hexagonal	$P6_322$	5.1161(11)	12.4817(27)	282.93(10)	4.3164
$K_2Ni_{1.5}Cu_{0.5}TeO_6$	Hexagonal	$P6_3/mcm$	5.2042(5)	12.0723(11)	283.16(39	4.9450
$K_2NiCuTeO_6$	Hexagonal	$P6_3/mcm$	5.1833(5)	11.8619(9)	276.01(3)	5.1022
$K_2MgCoTeO_6$	Hexagonal	$P6_3/mcm$	5.1807(6)	12.1783(15)	283.08(4)	4.5173
$K_2Ni_{1.5}Mn_{0.5}TeO_6$	Hexagonal	$P6_3/mcm$	5.1399(7)	12.0485(14)	275.65(4)	5.0295
$K_2NiMnTeO_6$	Hexagonal	$P6_3/mcm$	5.1319(7)	12.0715(15)	275.32(4)	5.0128

Supplementary Table 4. Atomic coordinates (*x*, *y*, *z*), occupancies (*g*), and isotropic atomic displacement parameters (U_{iso}) obtained by Rietveld refinement of conventional X-ray diffraction data for as-prepared $K_2Mg_2TeO_6$ indexed in the space group $P6_322$ (hexagonal) with lattice constants $a = b = 5.2863(5)$ Å, $c = 12.5388(2)$ Å, and $V = 303.45(6)$ Å^3 ($R_{Bragg} = 5.92$ %, $\chi^2 = 2.38$).

Atom	Wyckoff	*x*	*y*	*z*	*g*	U_{iso}
Mg1	2*b*	0	0	1/4	1	0.0082(10)
Mg2	2*d*	2/3	1/3	1/4	1	0.0082(10)
Te1	2*c*	1/3	2/3	1/4	1	0.0093(5)
O1	12*i*	0.6432(6)	-0.0252(4)	0.3452(1)	1	0.0154(42)
K1	12*i*	0.2408(37)	0.6534(29)	-0.0267(38)	0.0815(73)	0.0083(31)
K2	2*a*	0	0	0	0.1124(32)	0.0083(31)
K3	12*i*	0.7022(31)	0.0661(18)	-0.0064(22)	0.2341(58)	0.0083(31)

* *g* and U_{iso} denote the occupancy and isotropic thermal factor, respectively.

Supplementary Table 5. Atomic coordinates (*x*, *y*, *z*), occupancies (*g*), and isotropic atomic displacement parameters (U_{iso}) obtained by Rietveld refinement of conventional X-ray diffraction data for as-prepared $K_2Co_2TeO_6$ indexed in the space group $P6_322$ (hexagonal) with lattice constants $a = b = 5.2426(7)$ Å, $c = 12.4162(14)$ Å, and $V = 295.54(4)$ Å^3 ($R_{Bragg} = 5.78$ %, $\chi^2 = 2.14$).

Atom	Wyckoff	*x*	*y*	*z*	*g*	U_{iso}
Co1	2*b*	0	0	1/4	1	0.0078(5)
Co2	2*d*	2/3	1/3	1/4	1	0.0078(5)
Te1	2*c*	1/3	2/3	1/4	1	0.0088(4)
O1	12*i*	0.6381(7)	-0.0248(6)	0.3391(5)	1	0.0152(6)
K1	12*i*	0.2371(4)	0.6489(4)	-0.0271(14)	0.0543(28)	0.0081(4)
K2	2*a*	0	0	0	0.1056(21)	0.0081(4)
K3	12*i*	0.7051(4)	0.0642(8)	-0.0057(3)	0.2265(64)	0.0083(4)

Supplementary Table 6. List of feasible potassium-based cathode materials grouped according to their dimensionality (1D, 2D and 3D) and diffusion barriers obtained by BVEL. Selected potassium-based compounds with low diffusion barriers have been shown for brevity.

Compound	Lattice	Symmetry	Diffusion Barrier (eV)	Dimensionality	Reference ICSD
K_3CoO_2	Orthorhombic	*Pnma*	0.25	2D	73212
$K_{0.3}MnO_2$	Orthorhombic	*Ccmm*	0.27	2D	156080
K_2FeSiO_4	Cubic	F-43m	0.30	3D	434059*
$KFeSiO_4$	Tetragonal	$I4mm$	0.32	1D	73423
$K_2Ni_2TeO_6$	Hexagonal	$P6_3/mcm$	0.35	2D	434032*
$K_2CoNiTeO_6$	Hexagonal	$P6_3/mcm$	0.35	2D	434033*
$KFePO_4F$	Orthorhombic	$Pna2_1$	0.59	1D	39560
K_2NiO_2	Tetragonal	$I4/mmm$	0.80	3D	201891
$K_2CuP_2O_7$	Tetragonal	P-42$_1m$	0.91	2D	191282
K_2FeSiO_4	Orthorhombic	$Pca2_1$	0.92	1D	434055*
$KFeSi_2O_6$	Tetragonal	$I4_1/a$	1.13	1D	66922
$KMnPO_4$	Triclinic	P-1	1.17	1D	78840
K_2FeGeO_4	Orthorhombic	$Pca2_1$	1.24	1D	434060*
KVP_2O_7	Monoclinic	$P2_1/c$	1.25	1D	68625
$K_2NiP_2O_7$	Monoclinic	$P2_1$	1.25	2D	80978
$KFeP_2O_7$	Monoclinic	$P2_1/c$	1.27	1D	202814
$K_2FeP_2O_7$	Tetragonal	P-42$_1m$	1.30	2D	191281
$K_2CoV_2O_7$	Tetragonal	P-42$_1m$	1.45	2D	195619
$KFePO_4$	Monoclinic	$P2_1/n$	1.82	1D	415458
$K_2MnP_2O_7$	Monoclinic	$P2_1/n$	2.08	1D	74600
$KFeSiO_4$	Hexagonal	$P6_322$	2.13	1D	280085
$K_2CuP_2O_7$	Orthorhombic	*Pbnm*	2.16	1D	79916
$KMnVO_4$	Monoclinic	$P2_1/n$	2.19	1D	158975
$KNiPO_4$	Orthorhombic	$Pna2_1$	2.30	1D	81271
$KFeGeO_4$	Hexagonal	$P6_3$	2.59	2D	280084
$KFeO_2$	Orthorhombic	*Pbca*	2.68	1D	94467
$K_2FeB_2O_7$	Tetragonal	$P321$	3.38	1D	247415

Crystal structure depot (CSD) number indicated in asterisks are for new crystal structures deposited in the ICSD repository database.

Supplementary Table 7. Calculated BVEL activation energies for some selected potassium-based solid electrolyte materials grouped according to calculated BVEL diffusion barriers and their BVEL migration map dimensionality (1D, 2D and 3D).

Compound	Lattice	Symmetry	Diffusion Barrier (eV)	Dimensionality	Reference ICSD
K_2CdO_2	Orthorhombic	*Pbcn*	0.09	1D	25004
K_2ZnO_2	Orthorhombic	*Ibam*	0.12	1D	34603
K_4SiO_4	Monoclinic	$P2_1/c$	0.26	1D	66073
K_4GeO_4	Triclinic	$P\text{-}1$	0.31	1D	37271
$K_{0.72}In_{0.72}Sn_{0.28}O_2$	Hexagonal	$P\text{-}6m2$	0.32	2D	30062
$K_2Mg_2TeO_6$	Hexagonal	$P6_322$	0.33	2D	434061*
$KBiO_3$	Cubic	$Im\text{-}3$	0.75	2D	73746
K_2MgSiO_4	Orthorhombic	$Pca2_1$	0.81	1D	83226
$K_3Sc(MoO_4)_3$	Orthorhombic	$Pbc2_1$	0.91	1D	201281
$KMgPO_4$	Monoclinic	$P2_1/c$	1.04	1D	50926
$K_2MgV_2O_7$	Tetragonal	$P4_2/mmm$	1.05	2D	202823
$K_2CaP_2O_7$	Monoclinic	$P2_1/n$	1.69	1D	281572
K_2ZnGeO_4	Orthorhombic	$Pca2_1$	1.86	1D	85006
$KAl(MoO_4)_2$	Hexagonal	$P\text{-}3m1$	1.94	1D	28018
$K_2Mg_2(MoO_4)_3$	Orthorhombic	$P2_12_12_1$	2.02	1D	200931
$K_4Mg(WO_4)_3$	Triclinic	$P\text{-}1$	2.03	1D	194187
K_2CaPO_4F	Orthorhombic	*Pcmn*	2.52	1D	ref **[5]**
$K_3Na(SO_4)_2$	Hexagonal	$P\text{-}3m1$	5.57	2D	26018
$K_2Mg_2(SO_4)_3$	Cubic	$P2_13$	7.59	1D	100420
$K_2Ca_2Si_2O_7$	Hexagonal	$P6_3/m$	10.46	1D	180795

Crystal structure depot (CSD) accession number indicated in asterisks are for new crystal structures deposited in the ICSD repository database.

Supplementary Table 8. Ionic conductivity values reported for K-ion superionic conductors, showing performance comparison with $K_2Mg_2TeO_6$.

Compound	$\sigma_{573\ K}$ / mS cm^{-1} (300 ^{o}C (573 K))	$\sigma_{300\ K}$ / mS cm^{-1} (25±3 ^{o}C (298±3 K))	Bibliography
**$K_2Mg_2TeO_6$**	**38**	**~ 10^{-2}**	**This work**
$K_{2-2x}Mg_{1-x}Si_{1+x}O_4$	36		[6]
$K_{0.59}Mg_{0.53}Sb_{0.47}O_2$	21		[7]
$K_2O\cdot 5.2Fe_2O_3\cdot 0.8ZnO$	18		[8]
$K_{0.56}Ni_{0.52}Sb_{0.48}O_2$	16		[7]
$KFeO_2$	1		[9]
$KBiO_3$	10^{-2}		[10]
$K_{1+x}Ga_{11}O_{17+x/2}$ (x=0~0.3)	~1		[11]
$K_{0.405}Bi_{0.865}AsO_4$	1.607×10^{-4}		[12]
$K_5Sb_5P_2O_{20}$	3.162×10^{-3}		[13]
K_2SbPO_6	1×10^{-4}		[13]
$K_{2-2x}Fe_{2-x}P_xO_4$ (x=0.05~0.10)	7.1		[14]
$K_{1.9}Al_{1.95}V_{0.05}O_4$	1.38		[15]
$K_{1.9}Fe_{1.95}V_{0.05}O_4$	6.94		[15]
$K_{0.70}Zn_{0.35}Sn_{0.65}O_2$		7.5×10^{-3}	[16]
$K_{0.72}Sc_{0.72}Hf_{0.28}O_2$		4×10^{-5}	[17]
$K_{0.72}In_{0.72}Zr_{0.28}O_2$		1×10^{-4}	[17]
$K_{0.72}In_{0.72}Hf_{0.28}O_2$		3.5×10^{-4}	[17]
$K_{0.72}In_{0.72}Sn_{0.28}O_2$		5.6×10^{-4}	[17]
$K_{0.80}Zn_{0.40}Sn_{0.60}O_2$		1×10^{-3}	[11]
$K_4Nb_6O_{17}$		1×10^{-2}	[11]
$K_{1.8}(Li_{2.45}Sb_{5.55})O_{16}$		1	[18]
$K_{1.54}(Mg_{0.77}Ti_{7.23})O_{16}$		220	[19]
$KTiOAsO_4$		10^{-3}~ 10^{-6}	[20]

Supplementary Table 9. Volumetric energy densities attained by reported cathode materials for rechargeable potassium ion battery in comparison with new tellurate compounds detailed in this work. The achieved volumetric energy density was obtained based on the product of the attained capacity and the average voltage of the respective cathode material. Also note that the attained volumetric capacity is the product of the density of the active cathode material and the capacity attained.

Compound	K-half cell configuration	Attained capacity	Average voltage	Attained capacity (energy density)
		$mAhg^{-1}$	V *vs*. K^+ / K	mAh cm^{-3} (mWh cm^{-3})
P3-$K_{0.5}MnO_2$ [33]	$K_{0.5}MnO_2$/0.7M KPF_6 in EC/DEC (1:1 vol%)/K metal	100	2.5 ~2.7 (2.6)	454.7 (1182.2)
$K_{0.3}MnO_2$ [34]	$K_{0.3}MnO_2$/1.5M KFSI in EC/DMC /K metal	136	2.7	482.8 (1303.5)
P2-$K_{0.6}CoO_2$ [35]	$K_{0.6}CoO_2$/0.7M KPF_6 in EC/DEC (1:1 vol%)/K metal	80	2.7	352.9 (952.8)
$K_{2/3}Ni_{1/6}Co_{1/6}Mn_{2/3}O_2$ [36]	$K_{2/3}Ni_{1/6}Co_{1/6}Mn_{2/3}O_2$/0.8M KPF_6 in EC/DEC (1:1 vol%)/K	76.5	3.1	284.1 (880.7)
P2-$K_{0.41}CoO_2$ [37]	$K_{0.41}CoO_2$/1M KFSI in EC/DEC (1:1 vol%)/K metal	60	3	
Prussian blue (PB) [38]	$K_2MnFe(CN)_6$/saturated $KClO_4$ in PC /K metal	142	3.6	255.6 (920.2)
Prussian white (PW) [39]	PW / 0.8M KPF_6 in PC:4*wt* %FEC/K metal	110	3.2	201.3 (644.2)
PTCDA [40]	PTCDA/0.8M KPF_6 in EC/DEC (1:1 vol%)/K metal	122	2.5	207.4 (518.5)
$KVPO_4F$ [41]	$KVPO_4F$/1M KPF_6 in EC/PC/K metal	92	4.02	286.6 (1152.1)
$K_3V_2(PO_4)_3$ [42]	$K_3V_2(PO_4)_3$/0.8M KPF_6 in EC/DEC (1:1 vol%)/K metal	54	3.7	
$KVOPO_4$ [41]	$KVOPO_4$/1M KPF_6 in EC/PC/K metal	84	3.95	262.6 (1037.2)
KVP_2O_7 [43]	KVP_2O_7/0.5M KPF_6 in EC/DEC/K metal	60	4.2	181.0 (760.2)
$K_2CoNiTeO_6$	$K_2CoNiTeO_6$/0.5M KTFSI in Pyr_{13}TFSI/K metal	30	4.3	142.2 (611.6)
$K_2Ni_2TeO_6$	$K_2Ni_2TeO_6$/0.5M KTFSI in Pyr_{13}TFSI/K metal	70	3.6	325.3 (1171.2)
$K_2NiMgTeO_6$	$K_2NiMgTeO_6$/0.5M KTFSI in Pyr_{13}TFSI/K metal	34.7	3.98	139.9 (556.7)
$K_2NiZnTeO_6$	$K_2NiZnTeO_6$/0.5M KTFSI in Pyr_{13}TFSI/K metal	56.8	3.75	267.2 (1001.9)
$K_2Ni_{1.5}Mg_{0.5}TeO_6$	$K_2Ni_{1.5}Mg_{0.5}TeO_6$/0.5M KTFSI in Pyr_{13}TFSI/K metal	69	3.7	293.6 (1086.2)
$K_2Ni_{1.5}Zn_{0.5}TeO_6$	$K_2Ni_{1.5}Zn_{0.5}TeO_6$/0.5M KTFSI in Pyr_{13}TFSI/K metal	75.4	3.7	352.9 (1305.5)
$K_2Co_{0.5}Ni_{1.5}TeO_6$	$K_2Co_{0.5}Ni_{1.5}TeO_6$/0.5M KTFSI in Pyr_{13}TFSI/K metal	51.7	3.9	244.8 (954.6)
$K_2Co_{1.5}Ni_{0.5}TeO_6$	$K_2Co_{1.5}Ni_{0.5}TeO_6$/0.5M KTFSI in Pyr_{13}TFSI/K metal	8	3.6	35.6 (128.3)
$K_2Ni_{0.5}Mg_{1.5}TeO_6$	$K_2Ni_{0.5}Mg_{1.5}TeO_6$/0.5M KTFSI in Pyr_{13}TFSI/K metal	22.9	3.86	98.9 (381.5)
$K_2Ni_{1.5}Cu_{0.5}TeO_6$	$K_2Ni_{1.5}Cu_{0.5}TeO_6$/0.5M KTFSI in Pyr_{13}TFSI/K metal	37	3.68	183.0 (673.3)
$K_2N_{i1.5}Mn_{0.5}TeO_6$	$K_2Ni_{1.5}Mn_{0.5}TeO_6$//0.5M KTFSI in Pyr_{13}TFSI/K metal	38.8	3.63	195.1 (708.4)
$K_2NiCuTeO_6$	$K_2NiCuTeO_6$/0.5M KTFSI in Pyr_{13}TFSI/K metal	27	3.79	137.8 (522.1)
$K_2NiMnTeO_6$	$K_2NiMnTeO_6$//0.5M KTFSI in Pyr_{13}TFSI/K metal	4.65	3.25	23.3 (75.8)
K_4NiTeO_6	K_4NiTeO_6/0.5M KTFSI in Pyr_{13}TFSI/K metal	28.51	3.58	
K_4CoTeO_6	K_4CoTeO_6/0.5M KTFSI in Pyr_{13}TFSI/K metal	4.5	3.2	
$K_3Ni_2SbO_6$	$K_3Ni_2SbO_6$/0.5M KTFSI in Pyr_{13}TFSI/K metal	47.98	3.868	

*Co-based tellurates such as $K_2Co_2TeO_6$ and K_4CoTeO_6 were found to be electrochemically inactive.

Supplementary Table 10. Volumetric energy densities attained by other polyanion-based cathode materials for rechargeable potassium ion battery also evaluated in this work. Owing to the inherently low electronic conductivity of polyanion moieties, a higher carbon loading on the assembled composite electrode was used (namely, 70: 15:15 (active cathode material: carbon: binder)). Note also that the particle size was in the micrometric range, just as was observed in the tellurates, for all the polyanion compounds tested.

Compound	K-half cell configuration	Attained capacity	Average voltage	Attained capacity (energy density)
		$mAhg^{-1}$	V *vs*. K^+ / K	$mAh\ cm^{-3}$($mWh\ cm^{-3}$)
$KFePO_4F$	$KFePO_4F$/0.5M KTFSI in Pyr_{13}TFSI/K metal	×	×	×
$KFePO_4$	$KFePO_4$/0.5M KTFSI in Pyr_{13}TFSI/K metal	6.6	2.7	20.3 (54.8)
$KMnPO_4$	$KMnPO_4$/0.5M KTFSI in Pyr_{13}TFSI/K metal	2.5	2.92	7.5 (21.8)
$KCoPO_4$	$KCoPO_4$/0.5M KTFSI in Pyr_{13}TFSI/K metal	2.2	2.9	6.9 (20.1)
$KNiPO_4$	$KNiPO_4$/0.5M KTFSI in Pyr_{13}TFSI/K metal	2.2	2.94	7.2 (21.1)
$KMn_4(PO_4)_3$	$KMn_4(PO_4)_3$/0.5M KTFSI in Pyr_{13}TFSI/K metal	15.3	3.2	52.8 (168.8)
Beta-$VOPO_4$	Beta-$VOPO_4$/0.5M KTFSI in Pyr_{13}TFSI/K metal	10	3.55	32.4 (114.8)
$K_2VOP_2O_7$	$K_2VOP_2O_7$/0.5M KTFSI in Pyr_{13}TFSI/K metal	5	3.27	14.3 (46.7)
$KFeP_2O_7$	$KFeP_2O_7$/0.5M KTFSI in Pyr_{13}TFSI/K metal	2	2.8	6.2 (17.4)
$K_2FeP_2O_7$	$K_2FeP_2O_7$/0.5M KTFSI in Pyr_{13}TFSI/K metal	2	2.9	5.7 (16.5)
$K_2MnP_2O_7$	$K_2MnP_2O_7$/0.5M KTFSI in Pyr_{13}TFSI/K metal	1	2.9	3.2 (9.1)
Tetragonal $K_2CuP_2O_7$	$K_2CuP_2O_7$/0.5M KTFSI in Pyr_{13}TFSI/K metal	1.18	2.87	3.5 (10.0)
Orthorhombic $K_2CuP_2O_7$	$K_2CuP_2O_7$/0.5M KTFSI in Pyr_{13}TFSI/K metal	2.2	2.9	6.4 (18.5)
$K_2CoP_2O_7$	$K_2CoP_2O_7$/0.5M KTFSI in Pyr_{13}TFSI/K metal	×	×	×
$K_2NiP_2O_7$	$K_2NiP_2O_7$/0.5M KTFSI in Pyr_{13}TFSI/K metal	1.7	2.95	5.2 (15.4)
$Cu_2P_2O_7$	$Cu_2P_2O_7$/0.5M KTFSI in Pyr_{13}TFSI/K metal	1.8	2.63	7.5 (19.7)
$K_2CoV_2O_7$	$K_2CoV_2O_7$/0.5M KTFSI in Pyr_{13}TFSI/K metal	×	×	×
NASICON $Fe_2(SO_4)_3$	$Fe_2(SO_4)_3$/0.5M KTFSI in Pyr_{13}TFSI/K metal	19.8	3.52	60.7(213.5)
Yavapaiite $KFe(SO_4)_2$	$KFe(SO_4)_2$/0.5M KTFSI in Pyr_{13}TFSI/K metal	61	3.37	176.4 (594.5)
Langbeinite $K_2Mn_2(SO_4)_3$	$K_2Mn_2(SO_4)_3$/0.5M KTFSI in Pyr_{13}TFSI/K metal	2.5	2.91	7.6 (22.2)
Langbeinite $K_2Co_2(SO_4)_3$	$K_2Co_2(SO_4)_3$/0.5M KTFSI in Pyr_{13}TFSI/K metal	2.9	2.2	9.6 (21.1)
Langbeinite $K_2Ni_2(SO_4)_3$	$K_2Ni_2(SO_4)_3$/0.5M KTFSI in Pyr_{13}TFSI/K metal	32.8	2.1	110.5 (232.1)
Fedotovite $K_2Cu_3O(SO_4)_3$	$K_2Cu_3O(SO_4)_3$/0.5M KTFSI in Pyr_{13}TFSI/K metal	5	3.7	15.9 (58.8)
Alpha-$VOSO_4$	Alpha-$VOSO_4$/0.5M KTFSI in Pyr_{13}TFSI/K metal	19.6	2.2	66.0 (145.2)
$KCoSO_4F$	$KCoSO_4F$/0.5M KTFSI in Pyr_{13}TFSI/K metal	×	×	×
$Fe_2O(SO_4)_2$	$Fe_2O(SO_4)_2$/0.5M KTFSI in Pyr_{13}TFSI/K metal	×	×	×
$KCuSO_4F$	$KCuSO_4F$/0.5M KTFSI in Pyr_{13}TFSI/K metal	10	4.2	

*Hardly any capacity could be obtained from $K_2CoV_2O_7$, $K_2CoP_2O_7$, $KCoSO_4F$, $Fe_2O(SO_4)_2$, $KFeP_2O_7$, $KMnVO_4$ polyanion-based compounds.

Supplementary Table 11. Lattice parameters and the corresponding voltage of $K_2Ni_2TeO_6$ during galvanostatic charge (electrochemical K-ion extraction) and following discharge (K-ion reinsertion). The *a*-lattice parameter decreases whilst the *c*-lattice parameter increases upon K^+ extraction, as is expected, with a minimal volume change (*ca*. 2.18%).

**$K_{2-x}Ni_2TeO_6$ sample state of (dis)charge**	**Lattice**	**Symmetry**	**a_{hex} (Å)**	**c_{hex} (Å)**	**γ (°)**	**V (Å^3)**
Pristine (before cycling)	Hexagonal	$P6_3/mcm$	5.2606(1)	12.4669(3)	120	298.79(1)
Charged to 4 V	Hexagonal	$P6_3/mcm$	5.1740(7)	12.6192(6)	120	292.57(2)
Charged to 4.7 V	Hexagonal	$P6_3/mcm$	5.1547(7)	12.7026(8)	120	292.29(4)
Discharged to 3 V	Hexagonal	$P6_3/mcm$	5.2097(2)	12.5071(3)	120	293.96(2)
Discharged to 1.5 V	Hexagonal	$P6_3/mcm$	5.2596(4)	12.4418(3)	120	298.08(2)

Supplementary Table 12. Bader charge of each atomic species in $K_{2-x}Ni_2TeO_6$ (x = 0, 1 and 2) during galvanostatic charging (electrochemical K-ion extraction).

	Bader Charge (averaged value) / $|e|$		
Atom	x = 0	x = 1	x = 2
K	0.838	0.886	
Ni^{2+}	1.291 (1.692)	1.300 (1.666)	
Ni^{3+}		1.394 (1.048)	1.413 (1.072)
Te	5.714	5.710	5.712
O	-1.662	-1.549	-1.423

[a] Parentheses values are the magnetic moments μ_B.

Supplementary Note 1. Computational methodology for bond valency energy landscape (BVEL).

To gain insight on the K^+ ion migration channel dimensionality, bond-valency energy landscape (BVEL) method implemented in the BondSTR software with soft bond valency parameters [1] of the FullProf Suite [2] was employed and the diffusion pathways visualised with VESTA software [3]. A distance of 10 Å was taken into account as the Coulomb and Morse-type interaction potential ranges. This empirical method avails plausible diffusion pathways under an adjusted cut-off energy, typically the activation energy (E_a). Although the qualitative E_a values of BVEL have unclear physical meaning, their good correlation with those of density functional theory (DFT) [4], deems the BVEL approach a legitimate tool to judge as to whether it is worthwhile to further examine using more cost demanding computational simulations (*viz*., DFT or molecular dynamics (MD) *etc*.) including experiment. The calculated BVEL map drawn with the isosurface at E_a = 0.35 eV for $K_2Ni_2TeO_6$ is shown in **Supplementary Figure 10**. Bond valency calculations indicate diffusion of potassium ions through K1 and K2 sites in a rotary-like motion along the *ab* plane, depicting a two-dimensional circular K^+ diffusion pathway within the honeycomb structure akin to a rotary Wankel-type motor engine.

Supplementary Note 2. Computational methodology for density functional theory (DFT).

The dispersion-corrected density functional theory (DFT-D) calculations were performed using the generalised gradient approximation (GGA) with the Perdew-Burke-Ernzerhof (PBE) exchange-correlation functional [21] and projector augmented wave (PAW) pseudopotentials [22] as implemented in the Quantum-ESPRESSO (QE) Package [23]. DFT-D method can treat the interlayer van der Waals (vdW) interactions, correcting the PBE missing interaction tail; where Grimme's DFT-D2 [24] type of vdW correction was used. The PAW pseudopotentials were generated under the valency electron configurations explicitly represented as follows: O as $2s^2 2p^4$, Mg as $2s^2 2s^6 3s^2$, K as $3s^2 3p^6 4s^1$, Ni as $3d^8 4s^2$, and Te as $5s^2 5p^4$. A plane-wave cut-off of 60 Rydberg (Ry) for the kinetic energy and 500 Ry for the charge density were used to expand the wave functions. Full cell relaxation for all the target structures were conducted with a tolerance of 10^{-10} Ry for total energy and 10^{-3} eV/ Å for the residual forces on each ion. Spin-polarised GGA+U approach was applied for $K_{2-x}Ni_2TeO_6$ (x = 0, 1 and 2) with an antiferromagnetic ordering of Ni ions similar to that of $Na_2Ni_2TeO_6$ [25] adopting the $P6_3/mcm$ symmetry; where the effective Hubbard parameter U_{eff} (= $U - J$) for Ni d orbitals was taken to be 6 eV [26]. The fact that no geometrical information pertaining to the fully de-potassiated end-member Ni_2TeO_6 was available, a model was made with the central Ni/Te/O layer shifted *ca.* 1/3 along the *a* and *b*-axis direction in order to avoid direct attachment of oxygen atoms along the *c*-axis belonging to the different layers. Based on the optimal structures, the partial density of states (PDOS) around the Fermi level (E_F) applied for a Gaussian smearing parameter of 0.01 Ry, Bader charge density analysis (**Supplementary Table 12**) [27], and open-circuit voltage (OCV) as a reference to potassium metal, namely K^+ / K (see **Supplementary Figure 16**) were calculated using conventional protocols.

Supplementary Note 3. Computational methodology for DFT-based climbing-image nudged elastic band (DFT-NEB) method.

The activation energies (E_a) for $K_2Mg_2TeO_6$ were calculated using DFT-based climbing-image nudged elastic band (DFT-NEB) method including vdW correction with the GGA level of theory of the QE Package. A 10^{-2} eV / Å was chosen as the convergence threshold for ionic relaxation under the fixed lattice parameters. Prior to performing calculations, the vdW correction needed to reproduce the $P6_3/mcm$ symmetry, rather than $P6_322$ symmetry (as confirmed via experiment), was checked. Although similar formation energies for these symmetries were observed in $K_2Mg_2TeO_6$, the Na analogue ($Na_2Mg_2TeO_6$) tends to prefer more the $P6_322$ symmetry [28]. The self-diffusion coefficient, D and ionic conductivity, σ of K^+ ion in $K_2Mg_2TeO_6$ were estimated using Nernst-Einstein relation [29]:

$$\sigma T = (nq^2/k_B)\, D \quad (D = \rho\lambda^2 v^* \, exp\,(-E_a/k_B T)\ ^{30})$$

where, T is temperature, n the number density of charge carriers, q the movable ion's charge, k_B Boltzmann constant, ρ the geometric factor ≈1, λ the hopping distance of 3.4 (4.8) Å for $P6_3/mcm$ ($P6_322$) symmetry structures of $K_2Mg_2TeO_6$, and v^* the vibrational pre-factor ≈10^{13} Hz. Since it has been suggested from the results of the empirical-MD simulations that the high degree of movable ion-ion correlation prevailing necessitates a cooperative mechanism to be operative, such as that present in stoichiometric tellurium-oxides [31,32], it is sufficient to use the minimum unit-cell for DFT-NEB simulation as shown in **Supplementary Figure 12**. Here the K^+ ions at the top and bottom layers in the model were set to migrate in order to maintain the inversion symmetrical positions; thereby obviating the generation of the dipole moment within the cell.

Supplementary References

1. Adams, S. From bond valence maps to energy landscapes for mobile ions in ion-conducting solids. *Solid State Ionics* **177**, 1625 (2006).
2. Rodríguez-Carvajal, J. *FullProf Suite*; www.ill.eu/sites/fullprof/.
3. Momma, K. & Izumi, F. VESTA 3 for three-dimensional visualization of crystal, volumetric and morphology data. *J. Appl. Crystallogr*. **44**, 1272−1276 (2011).
4. Xiao, R., Li, H. & Chen, L. High-throughput design and optimization of fast lithium ion conductors by the combination of bond-valence method and density functional theory. *Sci. Rep*. **5**, 14227 (2015).
5. Daicho, H. et al. A novel red-emitting $K_2Ca(PO_4)F:Eu^{2+}$ phosphor with a large Stokes shift. *Chem. Commun.* **54**, 884−887 (2018).
6. Goodenough, J. B. & Whittingham, S. M. Solid State Chemistry of Energy Conversion and Storage. *Advances in Chemistry*; American Chemical Society: Washington, DC, 1977.
7. Smirnova, O. A. et al., Crystal structure, conductivity and reversible water uptake of new layered potassium antimonates $K_xL_{(1+x)/3}Sb_{(2-x)/3}O_2$ (L=Ni^{2+}, Mg^{2+}, Co^{2+}). *J. Solid State Chem*. **178**, 172 (2005).
8. Roth, W. I. & Romanczuk, R. J. Electrical Conductivity of Potassium Ferrite with the Beta-Alumina Structure. *J. Electrochem. Soc.* **116**, 975−980 (1969).
9. Proskurnina, N. V. et al., Ionic Conductivity in Ti-Doped $KFeO_2$: Experiment and Mathematical Modeling. *J. Phys. Chem. C* **121**, 21128−21135 (2017).
10. Nguyen, T. N., Giaquinta, D. M., Davis, W. M. & zur Loye, H. –C. Electrosynthesis of $KBiO_3$: A Potassium Ion Conductor with the $KSbO_3$ Tunnel Structure. *Chem. Mater*. **5**, 1273−1276 (1993).
11. http://www.icdd.com/membership/minutes/pdf/Ceramic-ION.pdf.
12. Falaha, C., Smida, Y. B., Ledoux-Rak, I. & Boughzala, H. Synthesis, crystal structure and ionic conductivity of a new open-framework arsenate $K_{0.405}Bi_{0.865}AsO_4$. *J. Alloys and Compounds* **653**, 321−326 (2015).
13. Wang, E. & Greenblatt, M. Ionic Conductivity of Potassium Phosphatoantimonates and Some of Their Ion-Exchanged Analogues. *Chem. Mater*. **3**, 542−546 (1991).
14. Burmakin, E. I. & Shekhtman, G. Sh. Potassium Ion Conducting $K_{2-2x}Fe_{2-x}P_xO_4$ Solid Electrolytes. *Inorg. Mater.* **44**, 882–885 (2008).
15. Burmakin, E. I., Nechaev, G. V., Antonov, B. D. & Shekhtman, G. Sh. Potassium-Conductive Solid Electrolytes in Systems $K_{2-2x}M_{2-x}V_xO_4$ (M = Al, Fe). *Russ. J. Electrochem.* **44**, 1171–1174 (2008).
16. Shukaev, I. L. & Butova, V. V. New P2 Compound with Brucite-Like Layers: Potassium Lithiostannate. *Inorg. Chem*. **51**, 4931 (2012).
17. Delmas, C., Fouassier, C., Reau, J. –M. & Hagenmuller, P. SUR DE NOUVEAUX CONDUCTEURS IONIQUES A STRUCTURE LAMELLAIRE. *Mat. Res. Bull.* **11**, 1081−1086 (1976).
18. Watelet, H., Besse, J. - P., Baud, G.& Chevalier, R. Determination of the structure and conductivity of a new hollandite-type compound: potassium (lithium, antimony) oxide ($K_{1.8}(Li_{2.45}Sb_{5.55})O_{16}$); characterization of a third order substructure. *Mater. Res. Bull.* **17**, 863 (1982).
19. Weber, H. –P. & Schulz, H. Ionic conduction in one dimension: A structural study of the hollandite $K_{1.54}Mg_{0.77}Ti_{7.23}O_{16}$ over the range $133 \leq T \leq 919$ K. *J. Chem. Phys.* **85**, 475 (1986).

20. Loiacono, G. M. et al., Optical properties and ionic conductivity of $KTiOAsO_4$ crystals *Appl. Phys. Lett.* **61**, 895 (1992).
21. Perdew, J. P., Burke, K. & Ernzerhof, M. Generalized Gradient Approximation Made Simple. *Phys. Rev. Lett*. **77**, 3865 (1996).
22. Dal Corso, A. Pseudopotentials periodic table: From H to Pu. *Comput. Mater. Sci.* **95**. 337−350 (2014).
23. Giannozzi , P. et al., QUANTUM ESPRESSO: a modular and open-source software project for quantum simulations of materials. *J. Phys.: Condens. Matter.* **21**, 395502 (2009).
24. Grimme, S. *J.* Semiempirical GGA-Type Density Functional Constructed with a Long-Range Dispersion Correction. *Comput. Chem.* **27**, 1787−1799 (2006).
25. Karna, S. K. et al., Sodium layer chiral distribution and spin structure of $Na_2Ni_2TeO_6$ with a Ni honeycomb lattice. *Phys. Rev.* B **95**, 104408 (2017).
26. Jain, A. et al., Formation enthalpies by mixing GGA and GGA +U calculations. *Phys. Rev.* B **84**, 045115 (2011).
27. Henkelman, G., Arnaldsson, A. & Jónsson, H. A fast and robust algorithm for Bader decomposition of charge density. *Comput. Mater. Sci.* **36**, 354−360 (2006).
28. Evstigneeva, M. A., Nalbandyan, V. B., Petrenko, A. A., Medvedev, B. S. & Kataev, A. A. A New Family of Fast Sodium Ion Conductors: $Na_2M_2TeO_6$ (M = Ni, Co, Zn, Mg). *Chem. Mater.* **23**, 1174−1181 (2011).
29. Boyce, J. B. & Huberman, B. A. SUPERIONIC CONDUCTORS: TRANSITIONS, STRUCTURES, DYNAMICS. *Phys. Rep.* **51**, 189 (1979).
30. Van der Ven, A., Bhattacharya, J. & Belak, A. A. Understanding Li Diffusion in Li-Intercalation Compounds. *Acc. Chem. Res.* **46**, 1216−1225 (2013).
31. Sau K. & Kumar, P. P. Ion Transport in $Na_2M_2TeO_6$: Insights from Molecular Dynamics Simulation. *J. Phys. Chem. C* **119**, 1651−1658 (2015).
32. Sau, K. Influence of ion–ion correlation on Na^+ transport in $Na_2Ni_2TeO_6$: molecular dynamics study. *Ionics* **22**, 2379−2385 (2016).
33. Kim, H. et al. Investigation of potassium storage in layered P3-type $K_{0.5}MnO_2$ cathode. *Adv. Mater.* **29**, 1702480 (2017).
34. Vaalma, C., Giffin, G. A., Buchholz, D. & Passerini, S. Non-Aqueous K-Ion Battery Based on Layered $K_{0.3}MnO_2$ and Hard Carbon/Carbon Black. *J. Electrochem. Soc.* **163**, A1295−A1299 (2016).
35. Kim, H. et al. K-ion batteries based on a P2-type $K_{0.6}CoO_2$ cathode. *Adv. Energy Mater.* **7**, 1700098 (2017).
36. Liu, C. et al. $K_{0.67}Ni_{0.17}Co_{0.17}Mn_{0.66}O_2$: a novel cathode material for potassium-ion battery. *Electrochem. Commun.* **82**, 150−154 (2017).
37. Hironaka, Y., Kubota, K. & Komaba, S. P2- and P3-K_xCoO_2 as an electrochemical potassium intercalation host. *Chem. Commun.* **53**, 3693−3696 (2017).
38. Xue, L. et al. Low-Cost High-Energy Potassium Cathode. *J. Am. Chem. Soc*. **139**, 2164–2167 (2017).
39. He, G. & Nazar, L. F. Crystallite size control of Prussian white analogues for non-aqueous potassium-ion batteries. *ACS Energy Lett.* **2**, 1122–1127 (2017).
40. Xing, Z. et al. A perylene anhydride crystal as a reversible electrode for K-ion batteries. *Energy Storage Mater.* **2**, 63−68 (2016).
41. Chihara, K., Katogi, A., Kubota, K. & Komaba, S. $KVPO_4F$ and $KVOPO_4$ toward 4 volt-class potassium-ion batteries. *Chem. Commun*. **53**, 5208−5211 (2017).

42. Han, J. et al. Investigation of $K_3V_2(PO_4)_3$/C nanocomposites as high-potential cathode materials for potassium-ion batteries. *Chem. Commun.* **53**, 1805–1808 (2017).
43. Park, W. B. et al. KVP_2O_7 as a robust high-energy cathode for potassium-ion batteries: pinpointed by a full screening of the inorganic registry under specific search conditions. *Adv. Energy Mater.* **8**, 1703099 (2017).
