# Supplementary material for: Rechargeable potassium-ion batteries with honeycomb-layered tellurates as high voltage cathodes and fast potassium-ion conductors
Source: Nat Commun. 2018 Sep 20;9:3823. doi: 10.1038/s41467-018-06343-6 (PMC6147795; doi:10.1038/s41467-018-06343-6)
Supplement: Supplementary file 3 — Description of Additional Supplementary files [file 41467_2018_6343_MOESM3_ESM.pdf]

Data 1 : "crystallographic information file for $K_2Ni_2TeO_6$"
Data 2: "crystallographic information for $K_2Mg_2TeO_6$"
